# A Multi-Strategy Adaptive Coati Optimization Algorithm for Constrained Optimization Engineering Design Problems

**DOI:** 10.3390/biomimetics10050323

**Published:** 2025-05-16

**Authors:** Xingtao Wu, Yunfei Ding, Lin Wang, Hongwei Zhang

**Affiliations:** 1 School of Electrical Engineering, Shanghai Dianji University, Shanghai 201306, China; wuxingt@st.sdju.edu.cn; 2 Amerson Biomedical (Shanghai) Co., Ltd., Shanghai 201318, China; 3 National Centre of Excellence for Food Engineering, Sheffield Hallam University, Sheffield S9 2AA, UK; h.zhang@shu.ac.uk

**Keywords:** multi-strategy adaptive coati optimization algorithm, CEC2017, engineering design optimization problems, Lévy flight, nonlinear step size inertia factor, coati vigilante mechanism

## Abstract

Optimization algorithms serve as a powerful instrument for tackling optimization issues and are highly valuable in the context of engineering design. The coati optimization algorithm (COA) is a novel meta-heuristic algorithm known for its robust search capabilities and rapid convergence rate. However, the effectiveness of the COA is compromised by the homogeneity of its initial population and its reliance on random strategies for prey hunting. To address these issues, a multi-strategy adaptive coati optimization algorithm (MACOA) is presented in this paper. Firstly, Lévy flights are incorporated into the initialization phase to produce high-quality initial solutions. Subsequently, a nonlinear inertia weight factor is integrated into the exploration phase to bolster the algorithm’s global search capabilities and accelerate convergence. Finally, the coati vigilante mechanism is introduced in the exploitation phase to improve the algorithm’s capacity to escape local optima. Comparative experiments with many existing algorithms are conducted using the CEC2017 test functions, and the proposed algorithm is applied to seven representative engineering design problems. MACOA’s average rankings in the three dimensions (30, 50, and 100) were 2.172, 1.897, and 1.759, respectively. The results show improved optimization speed and better performance.

## 1. Introduction

The exponential growth of technology has elevated the importance of optimization problems within a multitude of disciplines. As critical instruments for resolving these issues, optimization algorithms have evolved from their traditional iterations to sophisticated intelligent algorithms [1]. Early optimization techniques are predominantly based on mathematical formulations and analytical approaches, including linear and nonlinear programming, which prove to be effective for straightforward problems. However, these methods frequently falter in effectively optimizing complex, high-dimensional, and nonlinear optimization problems [2].

The surge in computing power and the development of big data technology have prompted researchers to investigate more adaptable and efficient optimization algorithms. This has led to the emergence of intelligent optimization algorithms [3]. By mimicking natural biological behaviors and group intelligence, these algorithms are capable of identifying approximate optimal solutions in the complex search space. Consequently, they exhibit a robust optimization performance and a high degree of adaptability [4].

The coati optimization algorithm (COA) [5], introduced by Dehghani in 2023, is a recently developed meta-heuristic algorithm that emulates the hunting behavior of coatis. Nevertheless, the initial population of COA is generated randomly, leading to a deficiency in diversity. In the hunting stage, the algorithm employs a random strategy, which lacks adaptability. Moreover, the behavior of evading natural enemies also relies on a random strategy. These factors contribute to an imbalance between COA’s global search and local optimization capabilities, predisposing it to become trapped in local optima, exhibiting limited global exploration, and demonstrating poor convergence accuracy. The analysis indicates that the COA algorithm, akin to other meta-heuristic algorithms (MA), inherits the common shortcomings associated with this class of algorithms [6].

The COA’s search process consists of two separate phases: exploration and exploitation. The former phase relates to the algorithm’s ability to navigate global search, a determinant of its ability to locate the optima. Conversely, the latter phase concerns the algorithm’s proficiency in navigating the local search space, which affects the rate at which the optimal values are produced. The COA’s performance is directly proportional to the balance achieved between exploration and exploitation. Nonetheless, adhering to the “No Free Lunch” theory [7], it is acknowledged that none of the algorithms can efficiently address all optimization challenges. A significant challenge with meta-heuristic algorithms is their tendency to get trapped locally, and most of them struggle to circumvent this pitfall [8].

As optimization algorithms have evolved, a plethora of enhancements has been suggested to improve their efficacy [9]. Shang S et al. [10] optimized the extreme learning machine using the improved zebra optimization algorithm. Wang C L et al. [11] developed a sound quality prediction model that incorporates extreme learning machine enhanced by fuzzy adaptive particle swarm optimization. Zhang et al. [12] introduced the chaotic adaptive sail shark optimization algorithm, which integrates the tent chaos strategy. Hassan et al. [13] put forth an improved butterfly optimization algorithm featuring nonlinear inertia weights and a bi-directional difference mutation strategy, along with decision coefficients and disturbance factors. Zhu et al. [14] proposed an adaptive strategy and a chaotic dyadic learning strategy implemented through the improved sticky mushroom algorithm. Yan Y et al. [15] proposed an On-Load Tap-Changer fault diagnosis method based on the weighted extreme learning machine optimized by an improved grey wolf algorithm. Gürses, Dildar et al. [16] used the slime mold optimization algorithm, the marine predators algorithm, and the salp swarm algorithm for real-world engineering applications. Dehghani, Mohammad et al. [17] used the spring search algorithm to solve the engineering optimization problems.

Additionally, the COA is attracting considerable interest. Jia et al. [18] introduced a sound-based search encirclement strategy to enhance the COA, yet they overlooked the optimization of the initial population’s generation. Zhang et al. [19] enhanced the COA by applying it to practical engineering issues, albeit employing only a straightforward nonlinear strategy. Barak [20] suggested integrating the COA with the grey wolf optimization algorithm for tuning active suspension linear quadratic regulator controllers. Baş et al. [21] proposed the enhanced coati optimization algorithm, a nonlinear optimization algorithm that improves upon the COA by balancing its exploitation and exploration capacities, although it does not address the resolution of imbalances through the optimization of the exploitation phase. Wang et al. [22] utilized the enhanced COA in the context of wind power forecasting applications. Mu G et al. [23] proposed a multi-strategy improved black-winged kite algorithm to select features. Zhou Y et al. [24] used an improved whale optimization algorithm in the engineering domain. Meng WP et al. [25] used a Q-learning-driven butterfly optimization algorithm to solve the green vehicle routing problem.

Through the above analysis, the problem is solved, the metaheuristic algorithm is slow to converge and easily falls into the local optimum, and the optimization speed and performance of the COA are improved. The proposal of a multi-strategy adaptive COA incorporates strategies such as Lévy flight, a nonlinear inertia step factor, and an enhanced coati vigilante mechanism to optimize the algorithm’s performance. The Lévy flight mechanism is employed during the initialization phase to populate the initial solution positions uniformly, thus generating high-quality starting solutions and enriching the solution population. Consequently, the problem of the COA’s initial solution, suffering from poor quality and uneven distribution, is addressed. During the exploration period, a nonlinear inertia weight parameter is incorporated to balance the local and global search abilities of the COA. Meanwhile, during the exploitation period, an enhanced coati vigilante mechanism is implemented to facilitate the COA’s capacity to get away from local optima.

The MACOA searching capability is validated through experimental studies that employ the IEEE CEC2017 benchmark functions. The MACOA is compared with 10 popular algorithms across various dimensions (30, 50, and 100), respectively. A comparative analysis of the convergence curves for all 12 algorithms across these dimensions, along with an examination of boxplots representing the outcomes from multiple runs and search history graphs, reveals that the MACOA demonstrates better optimization results over the other algorithms. To further study the engineering applicability of the MACOA, seven engineering challenges, including the design of a gear train, a reducer, etc., are used to test the ability of MACOA’s optimization. The analysis of the experiment results for these engineering design problems confirms the practical efficacy of the MACOA in optimizing practical engineering problems.

## 2. Basic Theory

The COA is inspired by the behavior of long-nosed coatis [5]. Each individual coati is a candidate solution. They have two natural behaviors in the hunting period: (1) behaviors when hunting for iguanas, and (2) behaviors when escaping from predators. It can be interpreted as two phases: exploration and exploitation.

### 2.1. Hunting for Iguanas (Exploration)

During the exploration phase, the coatis initiate a hunt and attack on the iguana, with some coatis climbing a tree in order to get close to the iguana. Other coatis wait beneath the tree to hunt the iguana once it falls to the ground. This strategy enables individual coatis to relocate to various locations within the search space, which showcases the global search capability of the COA within the problem space, i.e., exploration.

During the exploration phase, $x_{best}^{t}$ represents the position of the best individual in the population, corresponding to the iguana location. Half of the coatis will make their way up the tree, and the other half will stay in their original location. They will be waiting for the iguana to come down. The position of the first half is shown in (1):

(1) $$x_{i}^{t+1}(j)=x_{i}^{t}(j)+r\cdot (x_{best}^{t}(j)-RI\cdot x_{i}^{t}(j)),\;i=1,2,\cdot \cdot \cdot ,\frac{N}{2},j=1,2,\cdot \cdot \cdot ,M$$

where $x_{i}^{t}(j)$ expresses the position of an individual, *t* is defined as the number of the current iteration, and *r* is in [0, 1]. *RI* picks 1 or 2 randomly. *N* is considered the size of the population. *M* expresses the size of the dimension.

After the iguana’s fall, it is placed randomly. Then, the coatis, which stay on the ground, move through the space, searching for the iguana. The position is updated by (2) and (3) below:

(2) $$Iguana_{ground}^{t}(j)=lb_{j}+r\cdot (ub_{j}-lb_{j})$$

(3) $$x_{i}^{t+1}(j)=\left\{\begin{array}{l} x_{i}^{t}(j)+r\cdot (Iguana_{ground}^{t}(j)-I\cdot x_{i}^{t}(j)),\\ \;\;if\;fitness(Iguana_{ground}^{t})<fitness(x_{i}^{t})\\ x_{i}^{t}(j)+r\cdot (x_{i}^{t}(j)-Iguana_{ground}^{t}(j)),else \end{array}\right.\;i=\frac{N}{2}+1,\frac{N}{2}+2,\cdot \cdot \cdot ,N$$

where *lb_j_* and *ub_j_* express the lower and upper limits of the *j*-th dimensional variable. *fitness*(·) is the formula for calculating fitness. $Iguana_{ground}^{t}$ expresses the new position of the iguana after falling. $x_{i}^{t}(j)$ is the value of the *i*-th dimensional variable for the *i*-th individual under the current iteration.

If the updated location improves the value of the fitness, it is the optional location. Otherwise, the coati remains in its previous position, i.e., a greedy selection is performed in (4).

(4) $$x_{i}^{t+1}=\left\{\begin{array}{l} x_{i}^{t+1},if\;fitness(x_{i}^{t+1})<fitness(x_{i}^{t})\\ x_{i}^{t},else \end{array}\right.$$

### 2.2. Escaping from Predators (Exploitation)

During the exploitation phase, the updating of the coati’s location was modelled on the natural behavior of a coati escaping from a predator. A coati escapes when a predator comes. The action of the coati in one strategy brings it close to another safe position around its current position, which reflects the local search capability of the COA, i.e., exploitation.

During the exploitation phase, random positions are generated near every coati’s location, as shown in (5) and (6).

(5) $$lb_{j}^{local}=\frac{lb_{j}}{t},ub_{j}^{local}=\frac{ub_{j}}{t},t=1,2,\cdot \cdot \cdot ,T$$

(6) $$x_{i}^{t+1}(j)=x_{i}^{t}(j)-(1-2r)\cdot (lb_{j}^{local}+r\cdot (ub_{j}^{local}-lb_{j}^{local})),i=1,2,\cdot \cdot \cdot ,N$$

where *T* represents the maximum iteration count. *t* denotes the current number of iterations. $ub_{j}^{local}$ and $lb_{j}^{local}$ express the upper and lower bounds of the j-th dimensional variable, which are updated with each iteration. *r* is a random value between 0 and 1.

Finally, one more greedy choice is made, i.e., (4).

## 3. Proposed Algorithm

Although the COA is highly optimized, its initial population is generated randomly. Furthermore, COA employs a random strategy during the hunting phase, and its behavior in avoiding natural enemies is also contingent upon this random approach. These factors contribute to an imbalance between the global search capabilities and local optimization abilities of COA, making it susceptible to converging on local optima, exhibiting limited global exploration capacity, and demonstrating poor convergence accuracy. To address these issues, we propose the following heuristic strategies.

### 3.1. Chaos Mapping for Lévy Flight

Conventional random strategies generate populations with certain drawbacks, such as a lack of population diversity, and their randomness may lead to the possibility that certain areas are over-explored. Therefore, a mapping process for randomly generated populations is necessary.

The chaotic mapping mechanism is highly uncertain and sensitive. It can generate complicated and unpredictable dynamic behaviors to achieve a broader range of exploration in the search space [26].

Lévy flight is a special random walk model that describes movement patterns with long-tailed distributions [27]. The mapping is used in optimization algorithms to improve the randomness, which can assist the algorithm in more effectively exploring the solution space. Therefore, the global optimization capability is increased [28]. Lévy flights are introduced in the initialization process of the MACOA, as shown in (7)–(9).

(7) $$\alpha ⊗Levi(\beta )\sim 0.01\frac{u}{{\left|v\right|}^{-\beta }}\left(\vec{X}(t)-\vec{X_{\alpha }}(t)\right)$$

(8) $$\sigma_{u}={\left[\frac{\Gamma (1+\beta )\sin(\frac{\pi \beta }{2})}{\Gamma (\frac{1+\beta }{2})\beta \times 2^{\frac{\beta -1}{2}}}\right]}^{\frac{1}{\beta }},\sigma_{v}=1$$

(9) X(t+1)=X(t)+α⊗Levi(β)

where *X*(*t*) denotes the location of the i-th coati, and *α* is the weight of the control step. $u\sim N(0,\sigma_{u}^{2})$. $v\sim N(0,\sigma_{v}^{2})$. *β* is a constant, which is 1.5.

### 3.2. Nonlinear Inertia Step Size Factor

In the global optimization phase, premature convergence can hinder the algorithm’s ability to identify the global optimal solution. The incorporation of a nonlinear inertia step factor can mitigate the risk of premature convergence to local optima by dynamically adjusting the step size, thereby preserving the diversity of the population.

The introduction of a nonlinear inertia step size factor can greatly improve search efficiency and convergence performance, and the COA can dynamically adjust individuals’ search behavior. This dynamic adjustment mechanism effectively enhances the balance between exploration and exploitation. It also enhances adaptability and robustness.

Considering that updating a coati’s position is related to a coati’s current position, a nonlinear inertia step size factor is introduced. The factor adjusts the relationship between the coati’s position update and the current position information, depending on the individual coati’s position. Then, the factor is calculated by (10):

(10) $$\omega =\frac{{\left(\frac{t}{T}\right)}^{Cn}}{{\left(\frac{t}{T}\right)}^{Cn}+{\left(1-\frac{t}{T}\right)}^{Cn}}$$

where *Cn* is a constant greater than 1 to control the degree of nonlinearities, which is taken as 2 in this method.

Initially, the value of *ω* is small, resulting in the position updates being less influenced by the current position. This facilitates a broader search range for the algorithm and enhances its global exploration capability. As the search process progresses, the value of *ω* is increasing over time. The effect brought by the current coati position becomes larger, which assists in obtaining the optimal solution. Furthermore, it enhances the convergence speed as well as its local exploration ability.

The improved formula for modelling coati positions in the first stage is shown in (11):

(11) $$x_{i}^{t+1}(j)=\omega \cdot x_{i}^{t}(j)+r\cdot (x_{best}^{t}(j)-I\cdot x_{i}^{t}(j)),\;i=1,2,\cdot \cdot \cdot ,\frac{N}{2}$$

### 3.3. Coati Vigilante Mechanism

In the local optimal search phase, the algorithm usually focuses on a certain region for detailed search. The vigilante mechanism can assist the algorithm in escaping local optima by introducing random perturbations or altering the direction of the search to enhance the algorithm’s exploration.

In the sparrow search algorithm, when part of the sparrows search for food, some of them act as vigilantes, responsible for monitoring the security of their surroundings and sounding an alarm when a potential threat is detected.

This mechanism not only improves the survival rate of the group but also facilitates the rapid dissemination of information. The introduction of the vigilante mechanism enables the algorithm to cope with complex optimization problems more effectively. In this way, the COA can maintain a higher degree of flexibility and dynamism in exploring the solution space [29].

Introducing the sparrow vigilante mechanism in the exploitation phase enhances the vigilance ability of the COA to search within an optional range. The coatis on the outskirts of the population will swiftly relocate to seek a safe area when they realize there is danger. The coati located in the center will walk around randomly in order to get close to others in the population. The sparrow vigilant mechanism formula is shown in (12).

(12) $$X_{i,j}^{t+1}=\left\{\begin{array}{l} X_{best}^{t}+\beta \cdot \left|X_{i,j}^{t}-X_{best}^{t}\right|,if\;f_{i}>f_{g}\\ X_{i,j}^{t}+K\cdot (\frac{\left|X_{i,j}^{t}-X_{worst}^{t}\right|}{(f_{i}-f_{W})+\varepsilon }),if\;f_{i}=f_{g} \end{array}\right.$$

where $X_{best}^{t}$ represents the global optimal position in the current iteration, and β represents the step control parameter. β~N(0,1). K is randomly selected in [−1, 1]. $f_{i}$ is the fitness value. $f_{g}$ is the greatest global greatest fitness value, and $f_{w}$ is the worst one. ε is a very small constant.

Equation (12) can be optimized to address the problem of the global search capability. A dynamically adjusted step factor [30] is introduced, as shown in (13).

(13) $$X_{i,j}^{t+1}=\left\{\begin{array}{l} X_{best}^{t}+\beta (t)\cdot \left|X_{i,j}^{t}-X_{best}^{t}\right|,\;if\;f_{i}>f_{g}\\ X_{i,j}^{t}+K(t)\cdot (\frac{\left|X_{i,j}^{t}-X_{worst}^{t}\right|}{(f_{i}-f_{W})+\varepsilon }),\;if\;f_{i}=f_{g} \end{array}\right.$$

(14) $$\beta (t)=f_{g}-(f_{g}-f_{w})\cdot {\left(\frac{T-t}{T}\right)}^{1.5}$$

(15) $$K(t)=(f_{g}-f_{w})\cdot e^{-20\cdot \tan{\left(\frac{t}{T}\right)}^{2}}\cdot (2\cdot rand-1)$$

where β(t) is a dynamically adjusted step factor, as shown in (14). K(t) is a dynamically adjusted step factor, as shown in (15). rand∈[0,1].

The introduction of dynamic step factors β(t) and K(t) enables the algorithm to adjust the search behavior dynamically. At the beginning of the algorithm, it focuses on exploration, and the later phase focuses on exploitation. These optimizations improve the adaptability and robustness of the COA.

### 3.4. Multi-Strategy Adaptive Coati Optimization Algorithm

**Figure 1:**
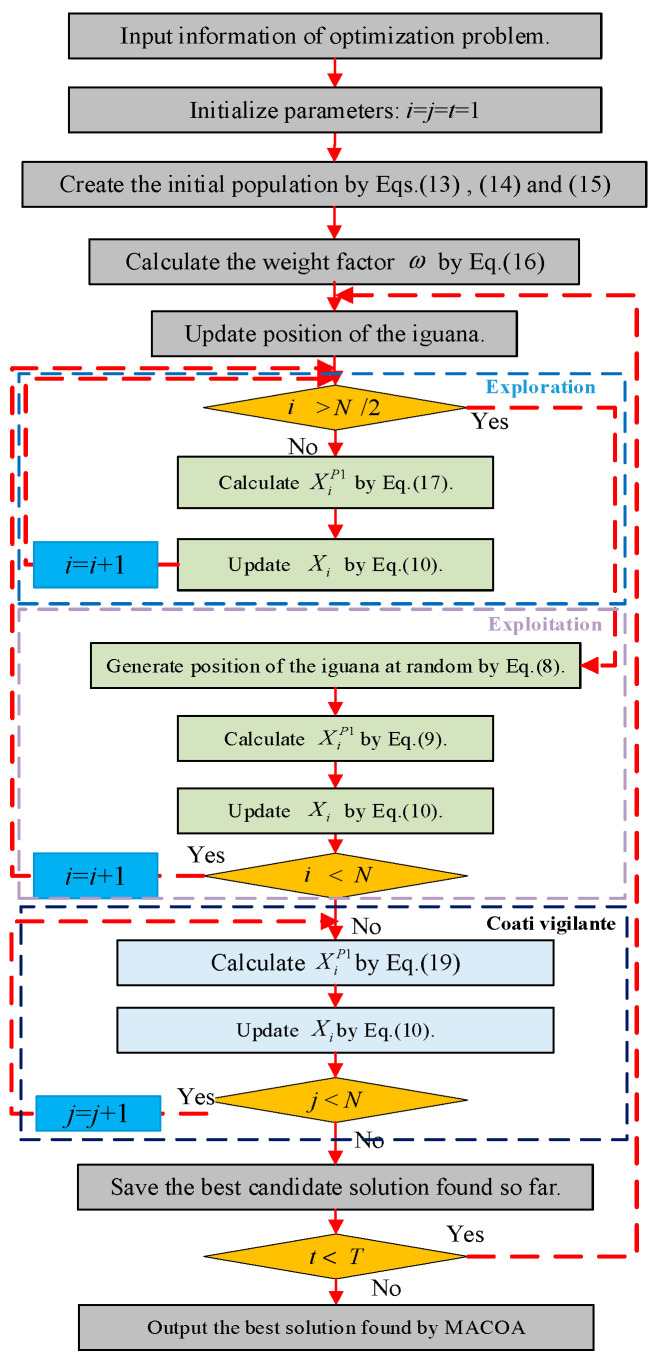
Flowchart of the MACOA.

The detailed flowchart of the MACOA is presented in Figure 1. The pseudo-code for MACOA is given in Table 1.

## 4. Experiments

Simulation studies and evaluations of the optimization efficiency for MACOA are presented. All experiments are conducted on an AMD 64-bit R7 processor operating at 3.20 GHz with 16 GB of RAM, utilizing MATLAB R2018a. This section uses tables that rank the optimal values produced by the algorithms, iteration curves from 10,000 iterations, and box plots from 50 experiments for statistical analysis.

### 4.1. Benchmark Functions and Compared Algorithms

Twenty-nine standard benchmark functions from the IEEE CEC-2017 [31] are utilized to test MACOA’s capability in addressing various objective functions. A comparison of MACOA’s performance with eleven well-known algorithms is performed in order to assess its quality in providing optimal solutions, namely COA, SABO [32], WSO [33], SCSO [34], GJO [35], TSA [36], WOA [37], GWO [38], TLBO [39], GSA [40], and PSO [41].

The results are presented through four metrics: mean, standard deviation (std), rank, and execution time (ET). The control parameter values for all compared algorithms are specified in Table 2.

### 4.2. Complexity Analysis

The complexity analysis of the algorithms was carried out using the problem definition and evaluation criteria of the CEC2017 Special Session and Competition on the Complexity of Single-Objective Constrained Numerical Optimisation Algorithms [42]. The steps are as follows:

(1) Calculate the system running time T_0_ by running the following test procedure:
  x = 0.05
  for i = 1:10,000
  x = x; x = x/2; x = x×x; x = sqrt(x);
  x = log(x); x = exp(x); x = x/(x + 2)
  end
(2) Calculate the complete computing time with 100,000 evaluations of the same D-dimensional function, i.e., *T*_1_.
(3) Calculate the complete computing time for the algorithm with 100,000 evaluations of the same D-dimensional function, i.e., *T*_2_.
(4) The complexity of the algorithm is reflected by (*T*_2_ − *T*_1_)/*T*_0_.

In this section, the algorithmic complexity analysis of MACOA, along with the other 11 algorithms in running the CEC2017 test function, is performed. In step (2), the maximum number of iterations is set to 10,000, and the number of dimensions is chosen to be 10. In step (3), the number of dimensions is set to 30. Table 3 lists the algorithmic complexity of each algorithm for running the CEC2017 functions. As can be seen from Table 3, the MACOA algorithm computes the function with increased time complexity compared to the COA algorithm, but the optimization performance is significantly improved over the COA algorithm. At the same time, from the point of view of the time complexity values of different algorithms for calculating the functions in CEC2017, the MACOA algorithm ranks in the middle in terms of the time complexity.

In the space complexity analysis, *pop* is the population size, *dim* is the dimension of the problem, and *Max* is the maximum number of iterations. From Table 4, it can be seen that MACOA does not improve the space complexity but improves the accuracy compared to COA.

### 4.3. Experimental Results and Analysis

CEC-2017 includes thirty standard benchmark functions of various types, as shown in Table 5. The test function F2 from the CEC-2017 is not used in this paper because of its unstable performance (as noted by other authors in their paper [19]). Complete information and details for these test functions can be found in Reference [31].

The proposed MACOA is subjected to 29 independent experiments at CEC-2017, each containing 200,000 FEs. Three dimensions of test functions are used in the experiments: 30, 50, and 100.

**Figure 2:**
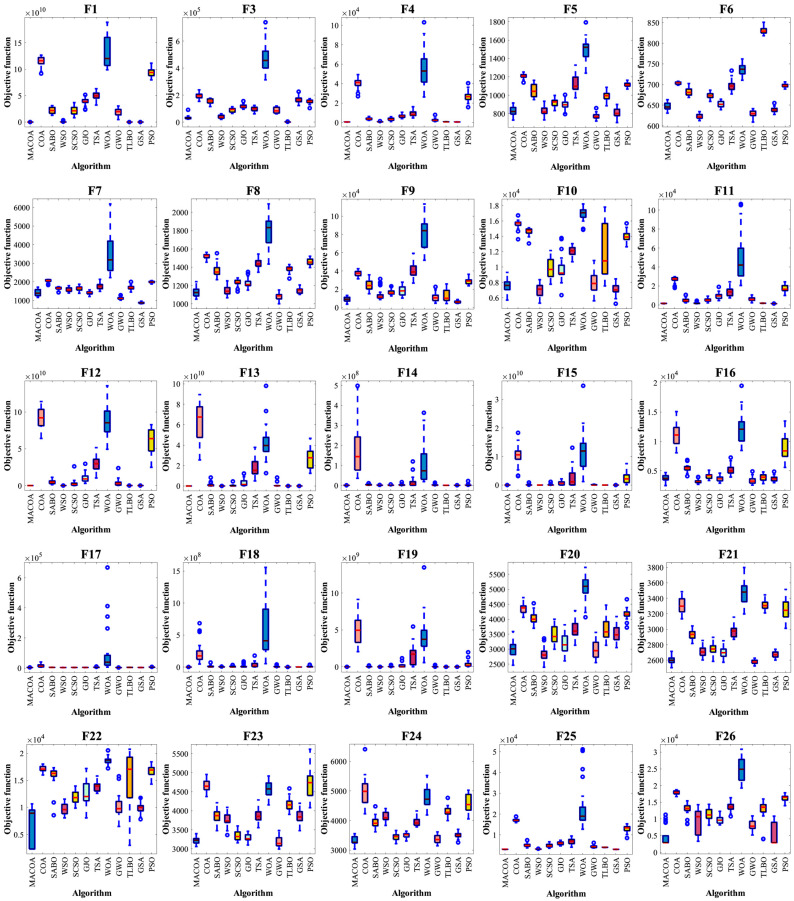

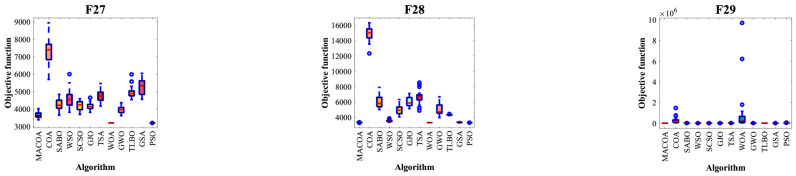
Box plots comparing MACOA and the other 11 algorithms based on the CEC-2017 (dimension m = 50).

The box plots in Figure 2 illustrate the distribution of results from 50 experiments. Based on the box-and-line plot, it can be seen that MACOA produces few outliers compared to other algorithms and achieves the optimal value for all algorithms in most of the tested functions. Therefore, MACOA has strong convergence and stability.

**Figure 3:**
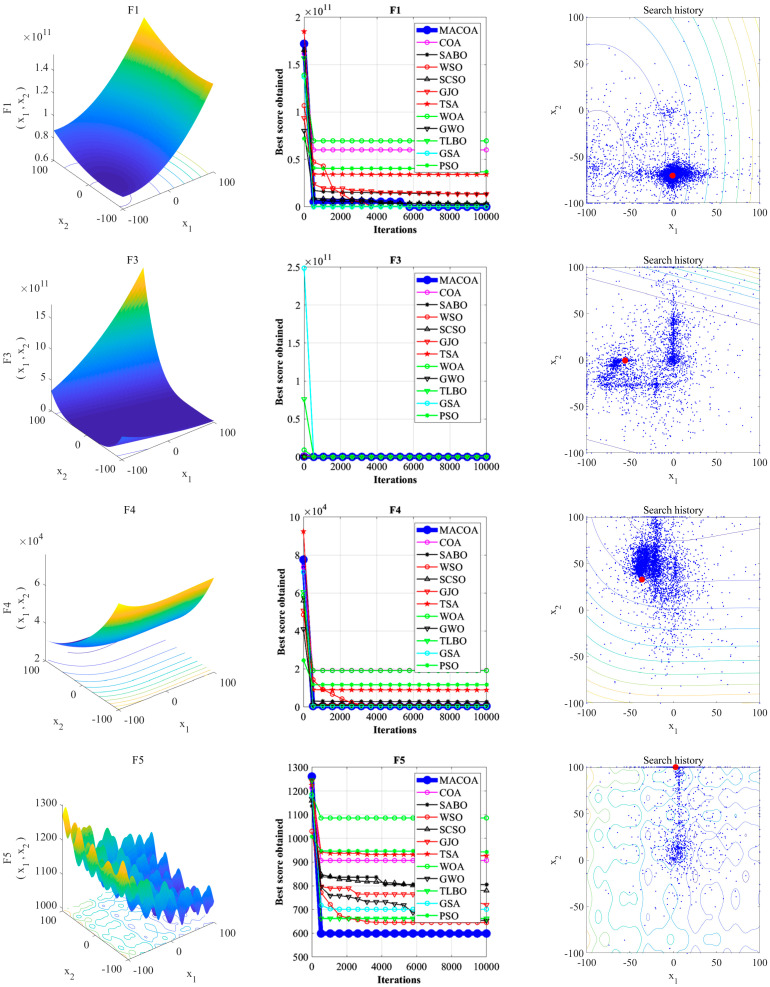

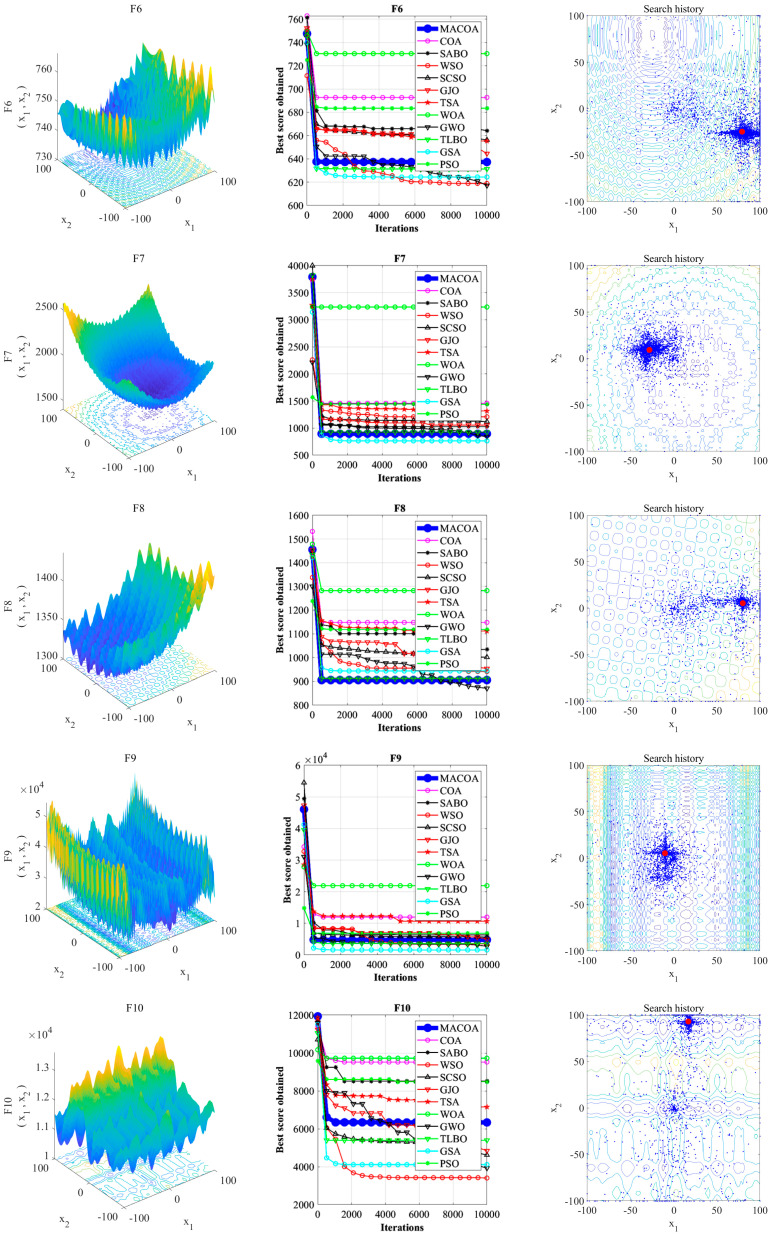

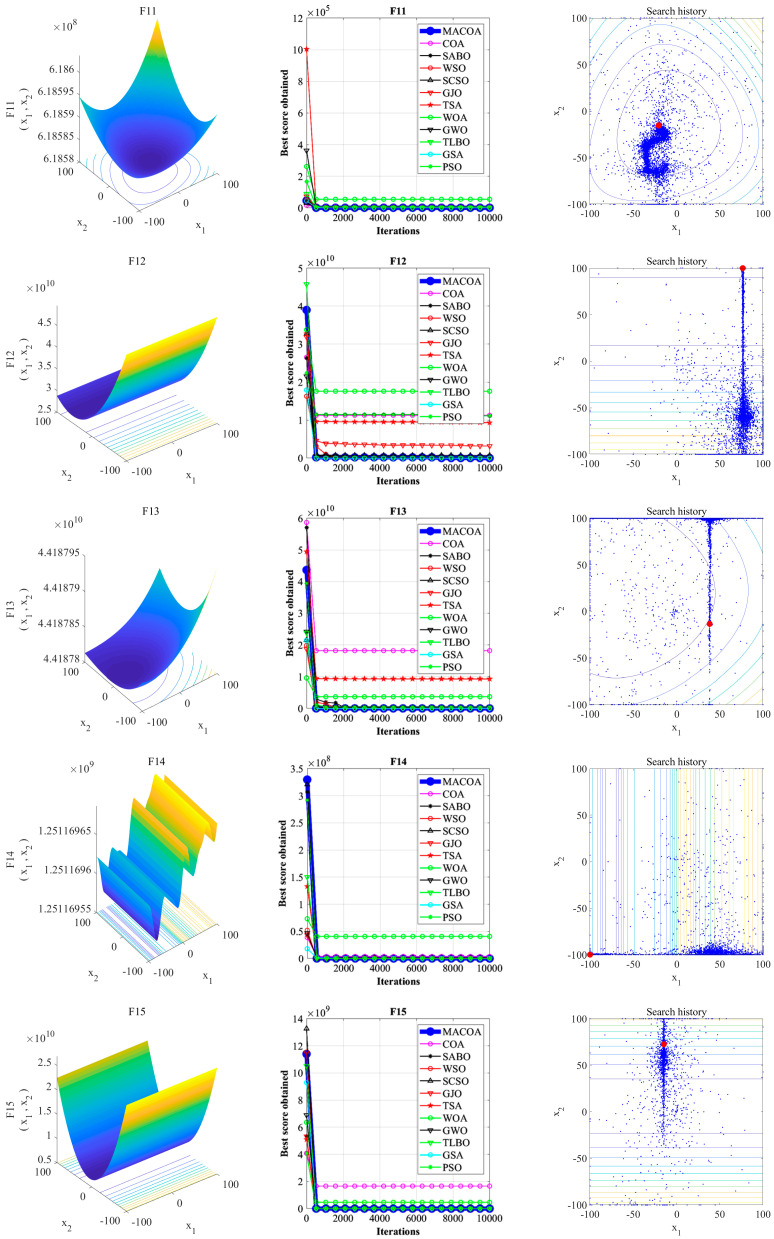

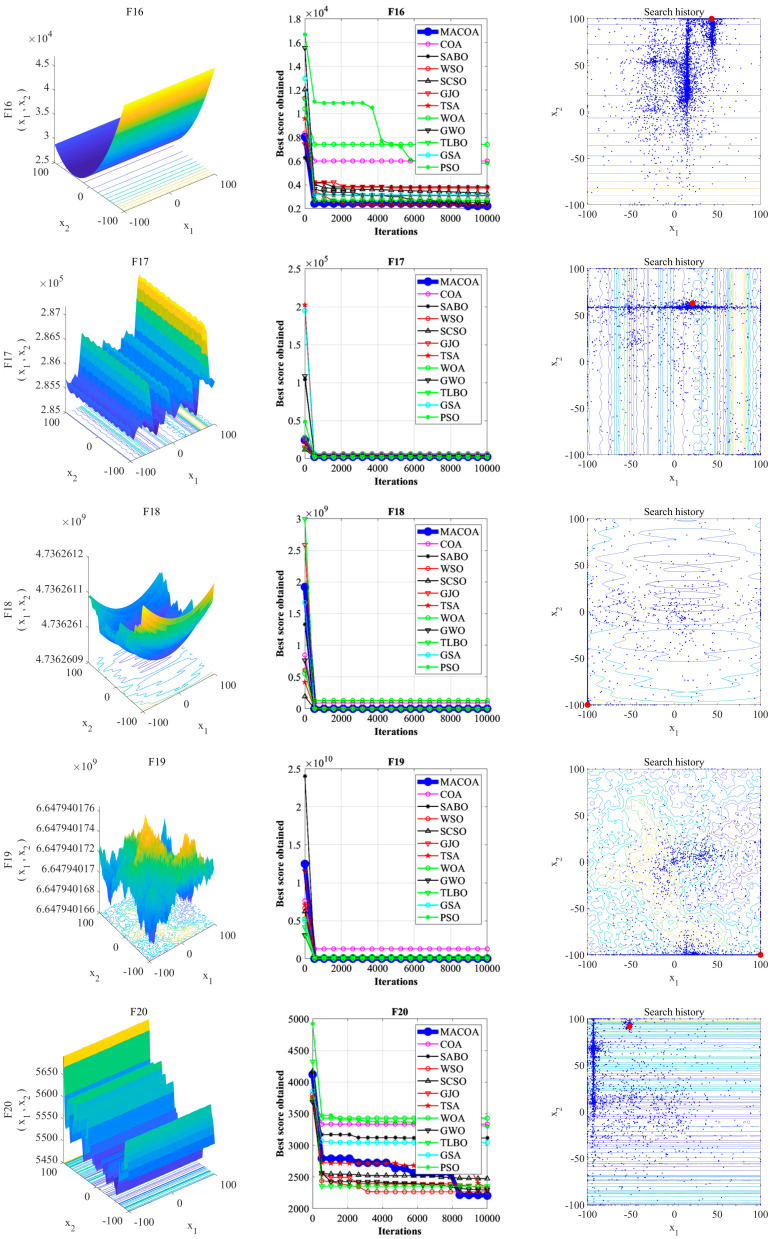

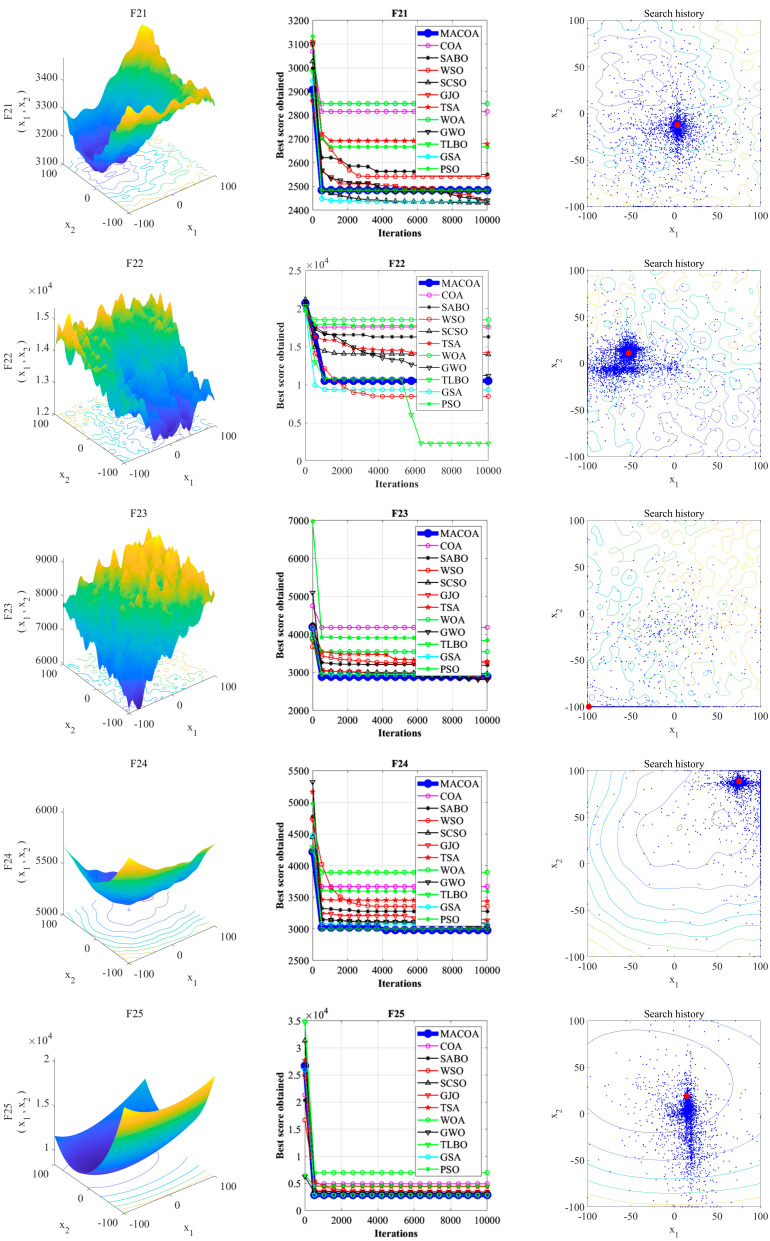

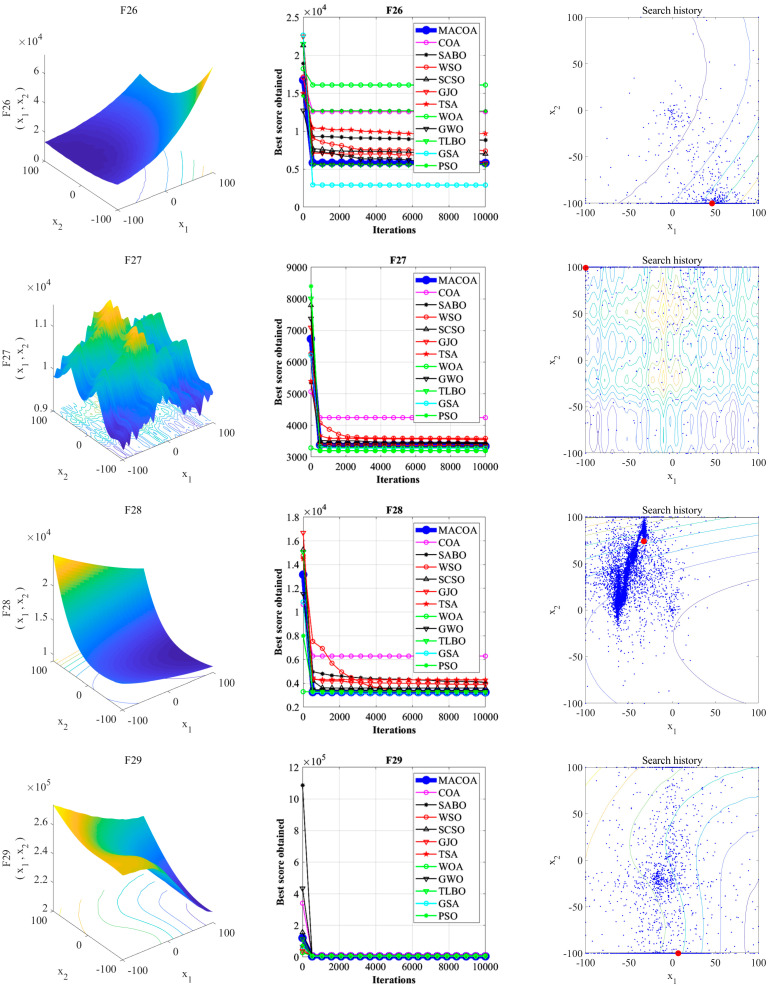
Iteration curves and search history of the proposed MACOA and the other 11 algorithms based on CEC2017 (dimension m = 30).

The 3D Surface Plots of CEC2017, iterative curves of the comparison algorithms, and search history plots are shown in Figure 3. According to the search history from Figure 3, the population distribution of MACOA is mostly located near the global optimal solution, and the overall convergence performance of the population is good.

**Figure 4:**
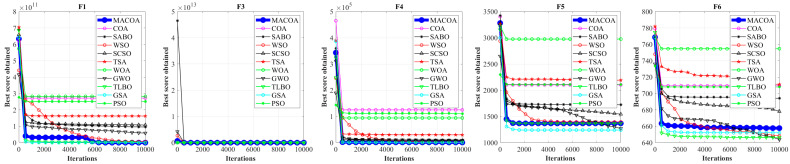

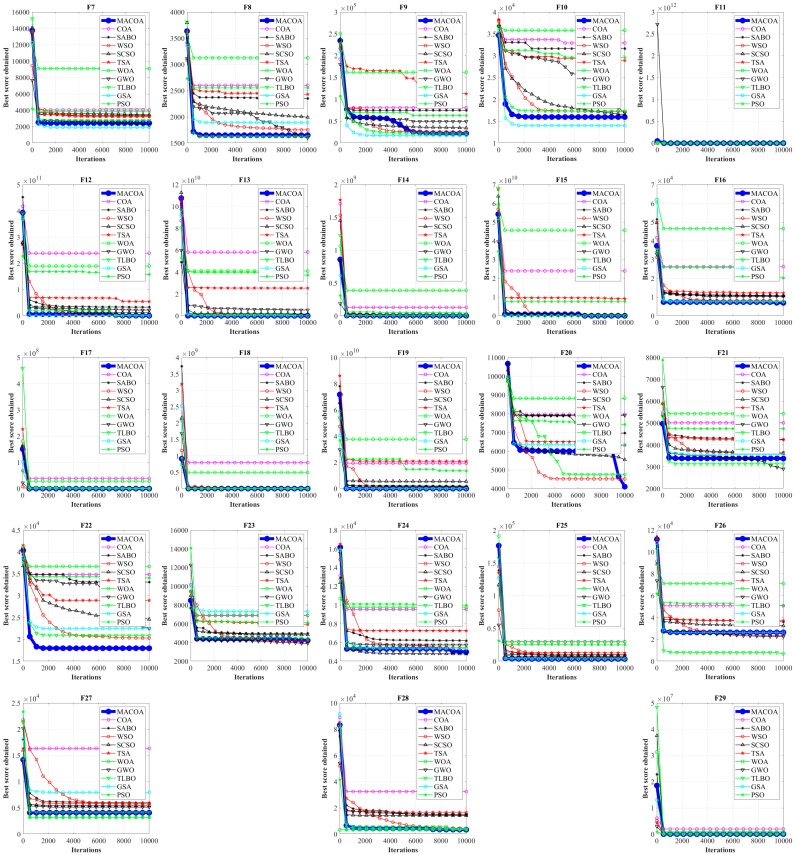
Iteration curves of MACOA and the compared algorithms based on the CEC-2017 (dimension m = 100).

Figure 3 and Figure 4 illustrate the convergence curves of MACOA and the compared algorithms after 10,000 iterations across the 29 benchmark functions from IEEE CEC2017. It is evident that MACOA exhibits a quicker convergence speed and superior convergence performance in comparison to other algorithms.

Additionally, the results are presented in Table 6, Table 7 and Table 8. The results for dimension 30 (m = 30) show that the MACOA is the best algorithm for solving F4, F10, F11, F22, F24~F26, F28, and F29 functions. The results of dimension 50 (m = 50) describe that MACOA is the best optimization algorithm for solving the F1, F4, F10, F11, F16, F18, F22~F26, and F29 functions. From the results for dimension 100 (m = 100), it can be obtained that MACOA is the best optimization algorithm for solving F1, F4, F10, F12, F14, F16, F17, F22~F26, F29, and F30 functions. Therefore, MACOA outperforms the comparison algorithms for most of the tested functions. Overall, MACOA works best in different dimensions (30, 50, and 100) of the CEC-2017 tested functions.

In comparison to the other 11 algorithms, the MACOA proposed has strong exploration, exploitation, and search capability. It shows improved performance compared to other optimization algorithms.

### 4.4. Ablation Experiment

In order to analyze the impact of different strategies on the performance of the algorithm, this section compares three strategies of MACOA through experimental analysis. In this section, the experiments are conducted using the test function of CEC2017, with all other parameters kept the same as before, and only the optimal value is used as the evaluation index. The results of the optimization study are shown in Table 9. L refers to Lévy flight, N refers to the nonlinear inertial step factor, and V refers to the coati vigilante mechanism.

From Table 9, it can be seen that the MACOA optimization with the introduction of the three strategies is the best. The second is COA + L + V with Levi flight and coati vigilante mechanism improvement. The third is nonlinear inertial step factor and vigilante mechanism improvement. All algorithms outperform the original COA. However, the results of COA + V and COA + L + N show that the coati vigilante mechanism works better than the other two strategies. In conclusion, all three improved strategies positively affect the original algorithm, proving the effectiveness of the heuristic strategy.

## 5. Engineering Problems

A benchmark suite of real-world non-convex constrained optimization problems and various established baseline results are utilized to analyze the engineering problems. In these constrained engineering problem designs, we use penalty terms as constraints. The optimization algorithm will find the global optimal solution under the constraints to achieve the constrained design. Problem difficulty within the benchmark is assessed using various evaluation criteria [43]. The algorithms COA, SABO, WSO, SCSO, GJO, TSA, SRS [44], MPA [45], and TLBO are included for comparative analysis, with each algorithm executed independently for 50 runs on all problems within the benchmark suite. Performance is evaluated based on feasibility rate (FR) and success rate (SR). FR represents the proportion of runs achieving at least one feasible solution within the maximum function evaluations. Meanwhile, SR denotes the proportion of runs where a feasible solution *x* satisfies f(x) − f(x*) ≤ 10^−8^ within function evaluations. This section uses tables of optimal, standard deviation, mean, median, worst, FR, and SR values generated by the algorithms, iteration curves generated by 10,000 iterations, box plots generated by 50 experiments, and search history for statistical analysis.

### 5.1. Three-Bar Truss Design Problem

**Figure 5:**
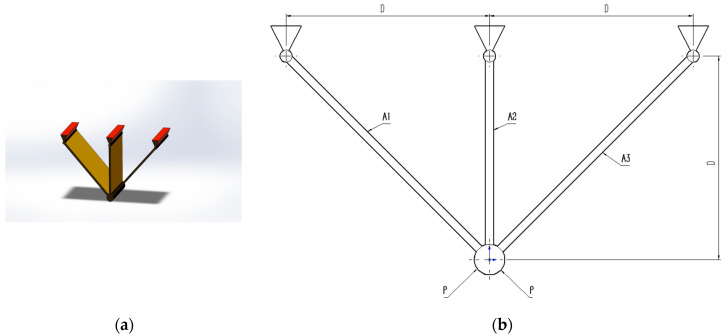
(**a**) Model diagram of the three-bar truss design problem. (**b**) Schematic of the three-bar truss design problem.

The three-bar truss design problem is to minimize the volume while satisfying the stress constraints on each side of the truss member. Figure 5 provides the geometry explanation. Within the benchmark suite, the problem features D = 2 decision variables, g = 3 inequality constraints, and h = 0 equality constraints. The optimal value of the objective function is known to be f(x*) = 2.6389584338 × 10^2^ [43].

The design problem for the three-bar truss can be outlined as follows:

Consider

(16) $$\vec{x}=[x_{1}\;x_{2}]=[A_{1}\;A_{2}]$$

Objective function:

(17) $$f(\vec{x})=(2\sqrt{2}x_{1}+x_{2})*l$$

Subject to

(18) $$g_{1}(\vec{x})=\frac{\sqrt{2}x_{1}+x_{2}}{\sqrt{2}x_{1}^{2}+2x_{1}x_{2}}P-\sigma \leq 0$$

(19) $$g_{2}(\vec{x})=\frac{x_{2}}{\sqrt{2}x_{1}^{2}+2x_{1}x_{2}}P-\sigma \leq 0$$

(20) $$g_{3}(\vec{x})=\frac{1}{\sqrt{2}x_{2}+x_{1}}P-\sigma \leq 0$$

where

(21) $$l=100\;\mathrm{cm},P=2\;{\mathrm{kN}/\mathrm{cm}}^{3},\sigma =2\;{\mathrm{kN}/\mathrm{cm}}^{3}$$

Boundaries:

(22) $$0\leq x_{1}\leq 1$$

(23) $$0\leq x_{2}\leq 1$$

**Figure 6:**
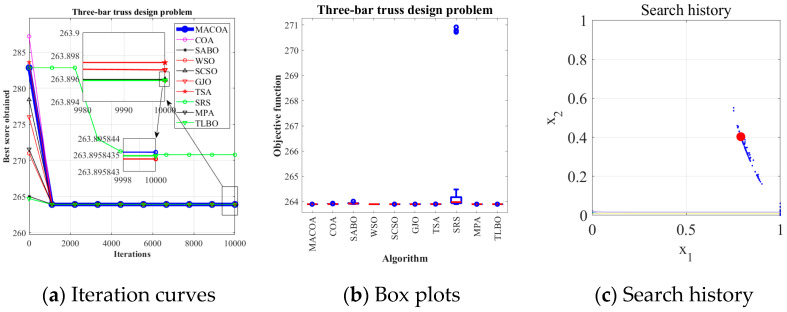
Iteration curves and box plots of the ten algorithms applied to the three-bar truss design problem.

From the experimental results in Table 10, it can be seen that MACOA has FR = 2 and SR = 10. These results show that MACOA’s FR score is second only to MPA, while its SR value is second only to MPA and TLBO. Moreover, the results of MACOA are significantly better than COA. Figure 6a illustrates the iteration process of the optimal solutions of the ten algorithms. The box-and-line plot is displayed in Figure 6b, and it can be seen that MACOA has strong stability. Figure 6c shows the search history, from which it can be seen that the search history of MACOA is concentrated around this neighborhood of the global optimal solution. Overall, these results show that MACOA outperforms COA.

### 5.2. Tension or Compression Spring Design Problem

The design of tension or compression springs represents a common optimization problem in mechanical engineering and structural design. The function of this device is to store and discharge energy.

**Figure 7:**
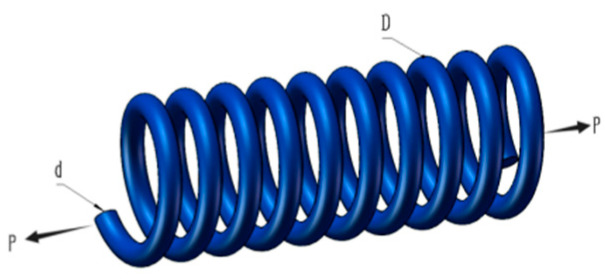
Schematic of the tension or compression spring design problem.

Therefore, a spring requires consideration of parameters during the design process. Within the benchmark suite, the problem features D = 3 decision variables, g = 4 inequality constraints, and h = 0 equality constraints. The optimal value of the objective function is known to be f(x*) = 1.2665232788 × 10^−2^ within the benchmark [43]. Figure 7 illustrates the pressure vessel configuration.

The design problem for tension or compression springs can be outlined as follows:

Consider

(24) $$x=[x_{1}\;x_{2}\;x_{3}]=[dDN]$$

Objective function:

(25) $$f(x)=(x_{3}+2)\times x_{2}\times x_{1}^{2}$$

Subject to

(26) $$g_{1}(x)=1-\frac{x_{3}\times x_{2}^{3}}{71785\times x_{1}^{4}}\leq 0$$

(27) $$g_{2}(x)=\frac{4\times x_{2}^{2}-x_{1}\times x_{2}}{12566\times (x_{2}\times x_{1}^{3}-x_{1}^{4})}+\frac{1}{5108\times x_{1}^{2}}-1\leq 0$$

(28) $$g_{3}(x)=1-\frac{140.45\times x_{1}}{x_{2}^{2}\times x_{3}}\leq 0$$

(29) $$g_{4}(x)=\frac{x_{1}+x_{2}}{1.5}-1\leq 0$$

Boundaries:

(30) $$0.05\leq x_{1}\leq 2.0$$

(31) $$0.25\leq x_{2}\leq 1.3$$

(32) $$2.0\leq x_{3}\leq 15.0$$

**Figure 8:**
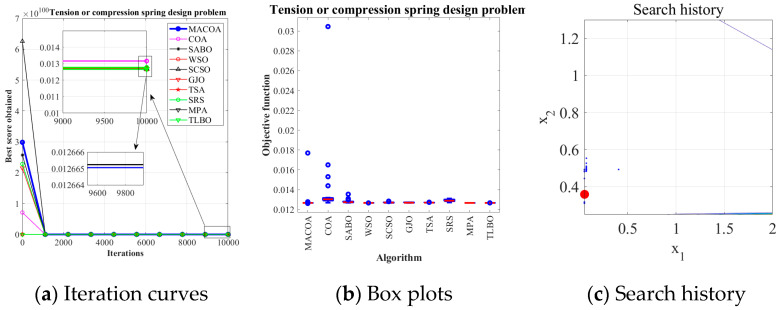
Iteration curves and box plots of the algorithms applied to the tension or compression spring design problem.

The experimental results in Table 11 show that MACOA has an FR of 98 and an SR of 98. These results indicate that MACOA has the highest FR among all the compared algorithms, and its SR is second only to WSO and MPA. Figure 8a illustrates the iteration process of the optimal solutions of the ten algorithms. The box-and-line plot is displayed in Figure 8b. It can be seen that MACOA has very few outliers. Figure 8c shows the search history. Although most of the search range lies on the boundary, it can be seen that most of the search history lies around the global optimum. Overall, these results show that MACOA outperforms COA.

### 5.3. Pressure Vessel Design Problem

**Figure 9:**
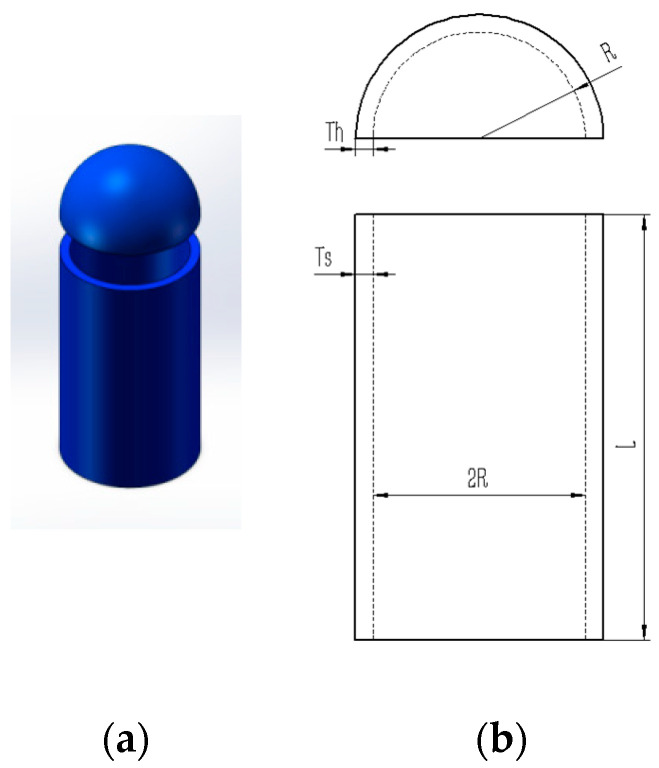
(**a**) Model diagram of the pressure vessel design problem. (**b**) Schematic of the pressure vessel design problem.

The pressure vessel design problem focuses on minimizing weight while maintaining structural integrity under high-pressure operating conditions. This involves optimizing design parameters, including material selection and wall thickness, within specified constraints to minimize the overall manufacturing costs. Within the benchmark suite, the problem features D = 4 decision variables, g = 4 inequality constraints, and h = 0 equality constraints. The optimal value of the objective function is known to be f(x*) = 5.8853327736 × 10^3^ [43]. Figure 9 illustrates the pressure vessel configuration. The design problem for the pressure vessel can be outlined as follows:

Consider

(33) $$\vec{x}=[x_{1}\;x_{2}\;x_{3}\;x_{4}]=[T_{S}\;T_{h}\;R\;L]$$

Objective function:

(34) $$\begin{array}{cc} f(\vec{x})= & 0.6224x_{1}x_{2}x_{3}+1.7781x_{2}x_{3}^{2}+\ldots \\ & 3.1661x_{1}^{2}x_{4}+19.84x_{1}^{2}x_{3} \end{array}$$

Subject to

(35) $$g_{1}(\vec{x})=-x_{1}+0.0193x_{3}\leq 0$$

(36) $$g_{2}(\vec{x})=-x_{3}+0.00954x_{3}\leq 0$$

(37) $$g_{3}(\vec{x})=-\pi x_{3}^{2}x_{4}+\frac{4}{3}\pi x_{3}^{3}+1296000\leq 0$$

(38) $$g_{4}(\vec{x})=-x_{4}-240\leq 0$$

Boundaries:

(39) $$0\leq x_{1}\leq 99$$

(40) $$0\leq x_{2}\leq 99$$

(41) $$10\leq x_{3}\leq 200$$

(42) $$10\leq x_{4}\leq 200$$

**Figure 10:**
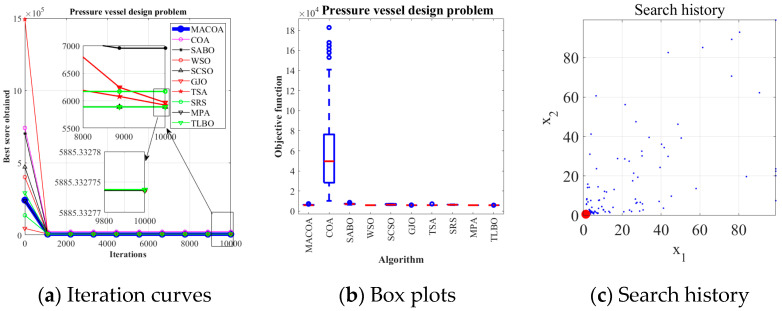
Iteration curves and boxplots of the ten algorithms applied to the pressure vessel design problem.

The results in Table 12 show that MACOA has the highest FR value among all the algorithms, with both FR and SR values of 98. Meanwhile, the SR value of MACOA is second only to TLBO and MPA. Figure 10a shows the iterative process for the optimal solution of the ten algorithms. Figure 10b, on the other hand, shows the box plots, from which it can be seen that the anomalies and quartiles of MACOA are concentrated with a certain degree of stability. Figure 10c shows the search history of MACOA, from which it can be seen that most of the search history of MACOA is concentrated near the global optimal solution region. These results show that MACOA can obtain the best performances.

### 5.4. Welded Beam Design Problem

**Figure 11:**
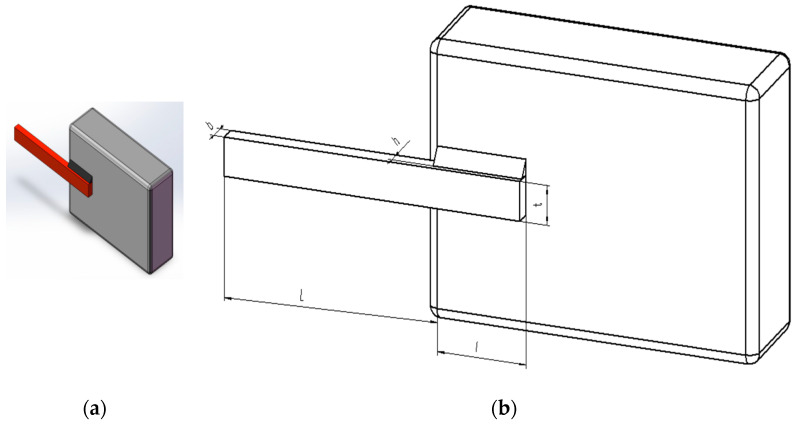
(**a**) Model diagram of the welded beam design problem. (**b**) Schematic of the welded beam design problem.

The welded beam design problem is to maximize structural performance while minimizing the beam’s weight by optimizing parameters such as weld dimensions, geometry, and placement, subject to specific constraints. Within the benchmark suite, the problem features D = 4 decision variables, g = 7 inequality constraints, and h = 0 equality constraints. The optimal value of the objective function is known to be f(x*) = 1.6702177263 [43]. Figure 11 describes the welded beam structure.

The design problem for the welded beam can be outlined as follows:

Consider

(43) $$x=[x_{1}\;x_{2}\;x_{3}\;x_{4}]=[h\;l\;t\;b]$$

Objective function:

(44) $$f(x)=1.10471x_{1}^{2}x_{2}+0.04811x_{3}x_{4}(14.0+x_{2})$$

Subject to

(45) $$g_{1}(\vec{x})=\tau (\vec{x})-\tau_{\max}\leq 0$$

(46) $$g_{2}(\vec{x})=\sigma (\vec{x})-\sigma_{\max}\leq 0$$

(47) $$g_{3}(\vec{x})=\delta (\vec{x})-\delta_{\max}\leq 0$$

(48) $$g_{4}(\vec{x})=x_{1}-x_{4}\leq 0$$

(49) $$g_{5}(\vec{x})=P-P_{C}(\vec{x})\leq 0$$

(50) $$g_{6}(\vec{x})=0.125-x_{1}\leq 0$$

(51) $$g_{7}(\vec{x})=1.10471x_{1}^{2}+0.04811x_{3}x_{4}(14.0+x_{2})-0.5\leq 0$$

where

(52) $$\tau (\vec{x})=\sqrt{{\left(\tau^{\prime }\right)}^{2}+2\tau^{\prime }\tau^{\prime \prime }\frac{x_{2}}{2R}+(\tau^{\prime \prime })},\tau^{\prime }=\frac{P}{\sqrt{2x_{1}x_{2}}},\tau^{\prime \prime }=\frac{MR}{J}$$

(53) $$M=P(L+\frac{x_{2}}{2}),R=\sqrt{\frac{x_{2}^{2}}{4}+{\left(\frac{x_{1}+x_{3}}{2}\right)}^{2}},\sigma (\vec{x})=\frac{6PL}{x_{4}x_{3}^{2}}$$

(54) $$J=2\left\{\sqrt{2}x_{1}x_{2}\left[\frac{x_{2}^{2}}{4}+{\left(\frac{x_{1}+x_{3}}{2}\right)}^{2}\right]\right\},\delta (\vec{x})=\frac{6PL^{3}}{Ex_{3}^{2}x_{4}}$$

(55) $$P_{c}(\vec{x})=\frac{4.013E\sqrt{x_{3}^{2}x_{4}^{6}/36}}{L^{2}}\left(1-\frac{x_{3}}{2L}\sqrt{\frac{E}{4G}}\right),$$

(56) $$P=6000lb,L=14in,\delta_{\max}=0.25in,E=30\times 10^{6}psi$$

(57) $$\tau_{\max}=13600psi\;and\;\sigma_{\max}=30000psi$$

Boundaries:

(58) $$0.1\leq x_{1}\leq 2$$

(59) $$0.1\leq x_{2}\leq 10$$

(60) $$0.1\leq x_{3}\leq 10$$

(61) $$0.1\leq x_{4}\leq 2$$

**Figure 12:**
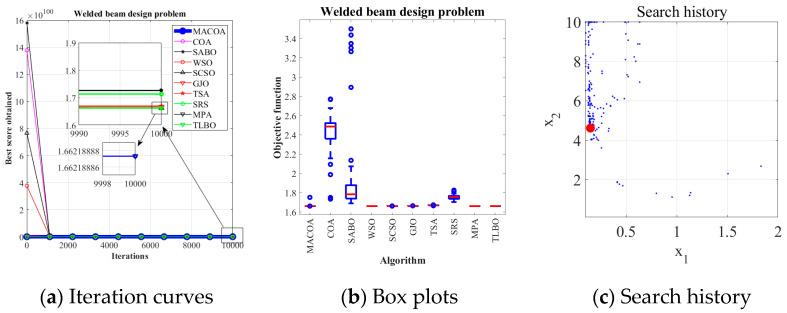
Convergence curves and box plots of the ten algorithms applied to the welded beam design problem.

The MACOA results in Table 13 show that both FR and SR are 84, which are significantly better than those of COA in both metrics. Figure 12a shows the iterative process of the optimal solutions found by the ten algorithms. Figure 12b shows the boxplots generated from 50 experiments, where MACOA is far superior to COA in terms of the number of anomalies and the median number of anomalies. Figure 12c shows the search history of MACOA, where most of the search histories are clustered around the lower bound. These results clearly show the superior performance of MACOA compared to COA.

### 5.5. Speed Reducer Design Problem

**Figure 13:**
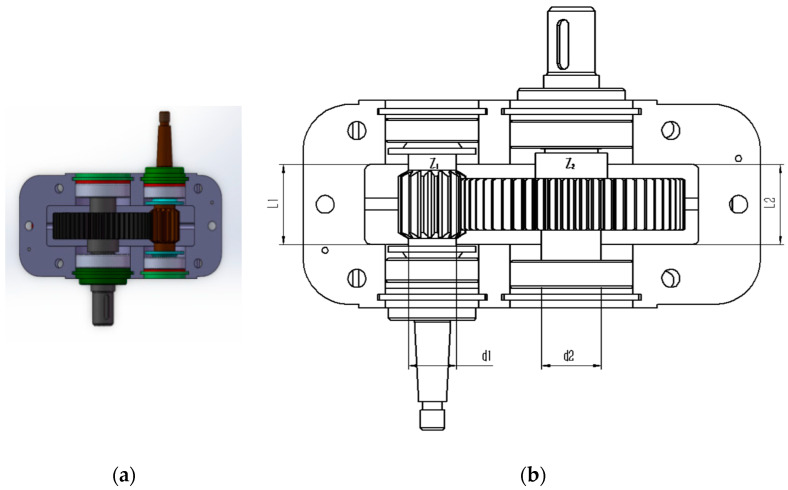
(**a**) Model diagram of the speed reducer design problem. (**b**) Schematic of the speed reducer design problem.

The speed reducer design problem is a well-known optimization challenge in engineering design. Within the benchmark suite, the problem features D = 7 decision variables, g = 11 inequality constraints, and h = 0 equality constraints. The optimal value of the objective function is known to be f (x*) = 2.9944 × 10^3^ [43]. Figure 13 illustrates the speed reducer configuration.

The design problem for the speed reducer can be outlined as follows:

Consider

(62) $$\vec{x}=[x_{1}\;x_{2}\;x_{3}\;x_{4}\;x_{5}\;x_{6}\;x_{7}]=[b\;m\;z_{2}\;l_{1}\;l_{2}\;d_{1}\;d_{2}]$$

Objective function:

(63) $$\begin{array}{l} f(X)=0.7854x_{1}x_{2}^{2}(3.3333x_{3}^{2}+14.9334x_{3}-43.0934)-\\ 1.508x_{1}(x_{6}^{2}+x_{7}^{2})+7.4777(x_{6}^{3}+x_{7}^{3})+0.7854(x_{4}x_{6}^{2}+x_{5}x_{7}^{2}) \end{array}$$

Subject to

(64) $$g_{1}(\vec{x})=\frac{27}{x_{1}x_{2}^{2}x_{3}}-1\leq 0$$

(65) $$g_{2}(\vec{x})=\frac{397.5}{x_{1}x_{2}^{2}x_{3}^{2}}-1\leq 0$$

(66) $$g_{3}(\vec{x})=\frac{1.93x_{4}^{3}}{x_{2}x_{6}^{4}x_{3}}-1\leq 0$$

(67) $$g_{4}(\vec{x})=\frac{1.93x_{5}^{3}}{x_{2}x_{7}^{4}x_{3}}-1\leq 0$$

(68) $$g_{5}(\vec{x})=\frac{\sqrt{{\left(745x_{4}/(x_{2}x_{3})\right)}^{2}+16.9\times 10^{6}}}{110x_{6}^{3}}-1\leq 0$$

(69) $$g_{6}(\vec{x})=\frac{\sqrt{{\left(745x_{5}/(x_{2}x_{3})\right)}^{2}+157.5\times 10^{6}}}{85x_{7}^{3}}-1\leq 0$$

(70) $$g_{7}(\vec{x})=\frac{x_{2}x_{3}}{40}-1\leq 0$$

(71) $$g_{8}(\vec{x})=\frac{5x_{2}}{x_{1}}-1\leq 0$$

(72) $$g_{9}(\vec{x})=\frac{x_{1}}{12x_{2}}-1\leq 0$$

(73) $$g_{10}(\vec{x})=\frac{1.5x_{6}+1.9}{x_{4}}-1\leq 0$$

(74) $$g_{11}(\vec{x})=\frac{1.1x_{7}+1.9}{x_{5}}-1\leq 0$$

Boundaries:

(75) $$2.6\leq x_{1}\leq 3.6$$

(76) $$0.7\leq x_{2}\leq 0.8$$

(77) $$17\leq x_{3}\leq 28$$

(78) $$7.3\leq x_{4}\leq 8.3$$

(79) $$7.8\leq x_{5}\leq 8.3$$

(80) $$2.9\leq x_{6}\leq 3.9$$

(81) $$5\leq x_{7}\leq 5.5$$

**Figure 14:**
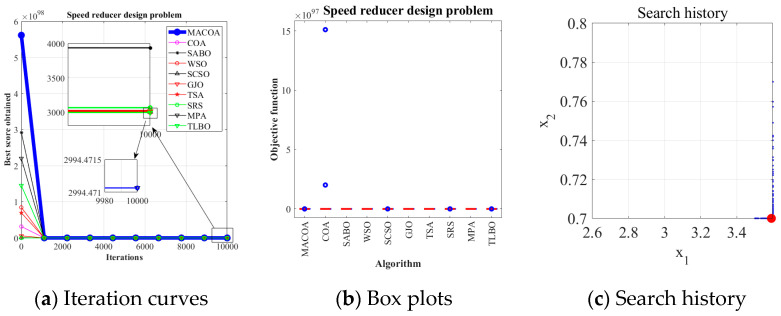
Iteration curves and box plots of the ten algorithms applied to the speed reducer design problem.

Table 14 shows that MACOA achieves FR = 46 and SR = 46, which are much higher than those of COA, and although WSO, MPA, and TLBO have slightly better FR and SR values, MACOA still outperforms other algorithms, including COA. Figure 14a shows the iterative process graphs for the optimal solutions of all algorithms. Figure 14b shows the boxplots of 50 experiments, demonstrating that MACOA has far fewer anomalies than COA. Figure 14c shows the search history of MACOA, with most of the search points clustered around the boundary and global optimal solutions. The above analysis can conclude that MACOA is significantly better than COA.

### 5.6. Gear Train Design Problem

**Figure 15:**
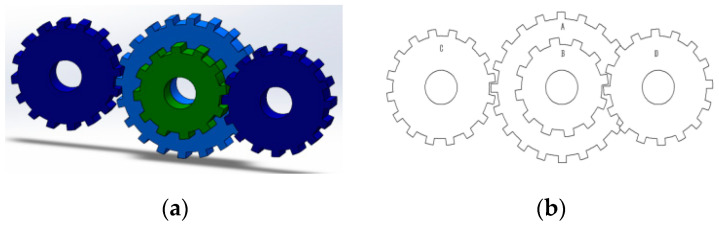
(**a**) Model diagram of the gear train design problem. (**b**) Schematic of the gear train design problem.

The problem of gear train design is a classic engineering design problem. The gear train design problem is proposed to minimize the gear ratio. Within the benchmark suite, the problem features D = 4 decision variables, g = 2 inequality constraints, and h = 0 equality constraints. The optimal value of the objective function is known to be f (x*) = 0 [43]. Figure 15 illustrates the gear train configuration.

The design problem for the gear train can be outlined as follows:

Consider

(82) $$\vec{x}=[x_{1}\;x_{2}\;x_{3}\;x_{4}]=[n_{A}\;n_{B}\;n_{C}\;n_{D}]$$

Objective function:

(83) $$f(\vec{x})={\left(\frac{1}{6.931}-\frac{x_{3}x_{2}}{x_{1}x_{4}}\right)}^{2}$$

Subject to

(84) $$g_{1}(\vec{x})=\frac{27}{x_{1}x_{2}^{2}x_{3}}-1\leq 0$$

(85) $$g_{2}(\vec{x})=\frac{397.5}{x_{1}x_{2}^{2}x_{3}^{2}}-1\leq 0$$

Boundaries:

(86) $$12\leq x_{1}\leq 60$$

(87) $$12\leq x_{2}\leq 60$$

(88) $$12\leq x_{3}\leq 60$$

(89) $$12\leq x_{4}\leq 60$$

**Figure 16:**
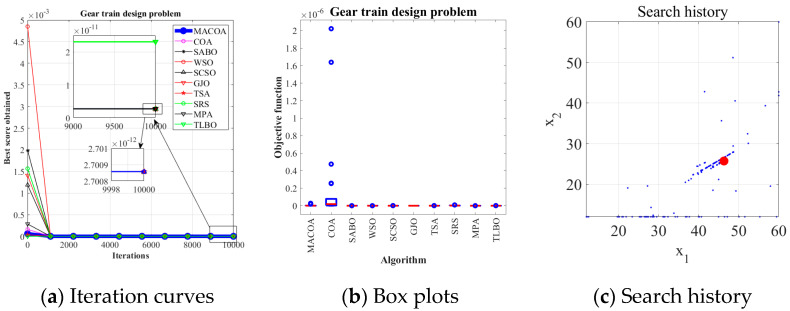
Iteration curves and boxplots of MACOA and the other algorithms for the gear train design problem.

Table 15 shows that the SR of MACOA is 92%, which greatly exceeds that of COA. Figure 16a and Figure 16b show the iterative process and the box plot distribution of the optimal solutions for all the algorithms, respectively. Figure 16c shows the search history of MACOA over 10,000 iterations, with most of the search regions located in the region where the global optimum is located. These results further highlight the improved performance of MACOA compared to COA.

### 5.7. Cantilever Beam Design Problem

**Figure 17:**
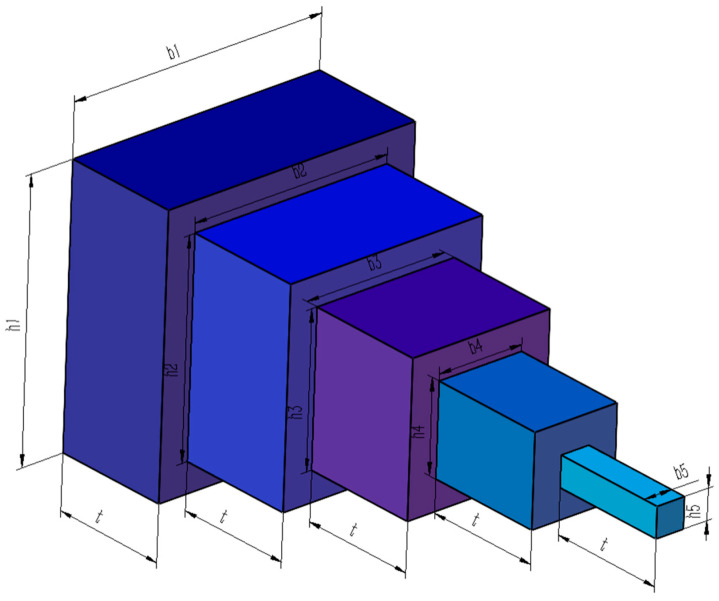
Schematic of the cantilever beam design problem.

The design of a cantilever beam is a classic engineering design problem. Within the benchmark suite, the problem features D = 5 decision variables, g = 1 inequality constraints, and h = 0 equality constraints. The established optimal objective function f (x*) is 1.34 [43]. Figure 17 illustrates the cantilever beam configuration.

The design problem for the cantilever beam can be outlined as follows:

Consider

(90) $$X=[x_{1}\;x_{2}\;x_{3}\;x_{4}\;x_{5}]$$

Objective function:

(91) $$f(X)=0.0624\left(\;x_{1}+x_{2}+x_{3}+x_{4}+x_{5}\right)$$

Subject to

(92) $$g_{1}(X)=\frac{61}{x_{1}^{3}}+\frac{37}{x_{2}^{3}}+\frac{19}{x_{3}^{3}}+\frac{7}{x_{4}^{3}}+\frac{1}{x_{5}^{3}}-1\leq 0$$

Boundaries:

(93) $$0.01\leq x_{1}\leq 100$$

(94) $$0.01\leq x_{2}\leq 100$$

(95) $$0.01\leq x_{3}\leq 100$$

(96) $$0.01\leq x_{4}\leq 100$$

(97) $$0.01\leq x_{5}\leq 100$$

**Figure 18:**
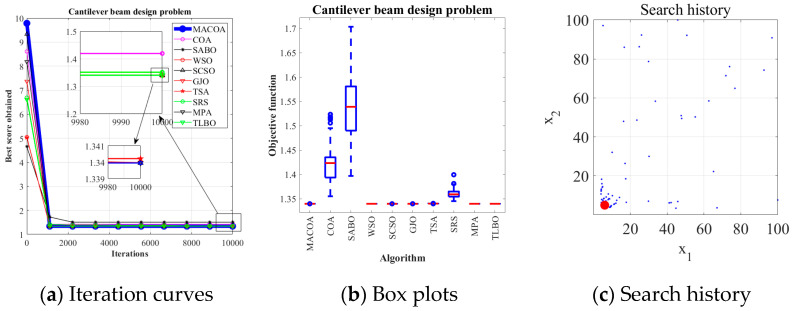
Iteration curves and box plots of the ten algorithms applied to the cantilever beam design problem.

Table 16 shows that MACOA has both FR and SR of 100. These metrics far exceed COA and are comparable to the performance of WSO, SCSO, MPA, and TLBO. Figure 18a shows the iterative convergence process of all the algorithms. Figure 18b shows the boxplot distribution, from which it can be seen that MACOA has better stability and convergence than COA. Figure 18c shows the search history of MACOA, where most of the searches are clustered around the global optimum. MACOA also demonstrates better population convergence. These results show that MACOA’s performance is very competitive, not only outperforming COA, but also being on par with other good optimization algorithms.

### 5.8. Summary of Engineering Problems

Examining the data gathered from tackling the seven engineering problems discussed above, it is clear that the MACOA algorithm excels compared to other algorithms regarding feasibility and success rate in most engineering problems. This demonstrates MACOA’s exceptional capability to solve constrained engineering problems.

## 6. Conclusions and Future Prospects

The challenge of slow convergence and the tendency of COA to converge to local optima are addressed in this paper. To mitigate these issues, MACOA is introduced, which integrates Lévy flight, nonlinear inertia weight factors, and the coati vigilante mechanism. The Lévy flight mechanism is introduced into the population initialization phase to improve the quality of initial solutions. Then, the nonlinear inertia weight factors are introduced in the exploration phase to improve COA’s global search capabilities and accelerate convergence. Additionally, the coati vigilante mechanism is implemented in the exploitation phase to enable the algorithm to quickly escape from local optima and address the imbalance between the exploration and exploitation capabilities of COA.

Experiments are conducted based on the IEEE CEC2017 test functions, comparing MACOA with 11 other popular algorithms across three dimensions. The analysis of convergence curves, boxplots, and search history results indicates that MACOA achieves the best performance on 9 test functions with an average ranking of 2.17 in the 30-dimensional experiment, 12 test functions with an average ranking of 1.90 in the 50-dimensional experiment, and 14 test functions with an average ranking of 1.76 in the 100-dimensional experiment. Overall, MACOA outperforms all compared algorithms in the CEC2017 test function experiments.

In application experiments, MACOA is tested on seven engineering problems. By analyzing the experimental results, it is clear that MACOA exhibits the best performance in four of these problems and outperforms COA in all application scenarios. Therefore, the proposed MACOA significantly improves the performance of COA and holds strong application value in constrained engineering optimization problems.

Although MACOA is overall effective at present, there are still some areas that need further improvement. In the standard test function experiments, MACOA did not perform well on some functions compared to other algorithms. In future work, we will continue our research on several optimization strategies, particularly the nonlinear strategy in this study, where the adaptive parameters change with iterations. In addition, we are working on the challenges of its integration with other complex disciplinary issues. These efforts will further validate the adaptability and effectiveness of MACOA in different domains.

## Figures and Tables

**Table 1:** Pseudo-code of MACOA.

**Start MACOA.**
Input the optimization problem information.
Set the number of iterations *T* and the number of coatis *N*.
Initialization of coatis and evaluation of the objective function for the population using (7)–(9).
For t = 1:*T*
Update location of the iguana based on the location of the best member of the population.
**Phase 1: Exploration Phase**
Calculate the weighted factor *ω* using (10)
For i = 1:[*N/2*]
Calculate new position for the i-th coati by (11).
Update position of the i-th coati using (4).
End for
for i = *N/2* +1:*N*
Calculate random position for the iguana using (2).
Calculate new position for the i-th coati using (3).
Update position of the i-th coati using (4).
End for
**Phase 2: Exploitation Phase**
For i = 1:*N*
Calculate the new position for the i-th coati using (13).
Update the position of the i-th coati using (4).
End for
Save the best candidate solution found so far
End for
**Output** of the best obtained solution by MACOA for given problem.
**End MACOA.**

**Table 2:** Control parameter values for the algorithms being compared.

Alg.	Parameter	Value	Alg.	Parameter	Value
COA	r: random number	[0, 1]	WOA	a: Convergence parameter	Linear reduction from 2 to 0.
	I: random number	{0, 1}		r: random vector	[0, 1]
SABO	v: random vector	[1, 2]		l: random number	[−1, 1]
	ri: random number	ri obeys a normal distribution	GWO	Convergence parameter (a)	Linear reduction from 2 to 0.
WSO	fmin	0.07	TLBO	TF: teaching factor	TF = round [(1 + rand)]
	fmax	0.75		Random number	[0, 1]
	τ	4.11	GSA	Alpha	20
	a0	6.25		Rpower	1
	a1	100		Rnorm	2
	a2	0.0005		G0	100
SCSO	r_G_	Linear reduction from 2 to 0.	PSO	Topology	Fully connected.
	S_M_	2		Cognitive constant	C1 = 2
GJO	c1	1.5		Social constant	C2 = 2
	E_0_: random number	[−1, 1]		Inertia weight	Linear reduction from 0.9 to 0.1.
	β	1.5		Velocity limit	10% of the dimensions range of the variables.
TSA	Pmin	1	WOA	a: Convergence parameter	a: Linear reduction from 2 to 0.
	Pmax	4		r: random vector	[0, 1]
	c1, c2, c3	Random in [0, 1]		l: random number	[−1, 1]

**Table 3:** Time complexity comparison between MACOA and the other 11 algorithms based on the CEC-2017.

	MACOA	COA	SABO	WSO	SCSO	GJO	TSA	WOA	GWO	TLBO	GSA	PSO
F1	852.5946	879.1897	509.3283	444.0638	7430.2372	1488.9901	981.1678	440.2204	951.0089	1595.0824	1734.3884	894.0729
F3	1020.6622	1022.7472	727.0783	551.5746	7199.4453	1278.7816	863.0205	350.4119	845.6234	1071.4026	1526.6804	770.3774
F4	932.7085	920.0671	658.0728	608.8970	7041.9431	1156.2279	829.5889	388.6270	1055.0448	1044.7861	1512.6196	772.6830
F5	1310.3827	1349.9389	977.0681	635.2419	7280.4222	1490.5998	1017.1022	578.9648	1114.7616	1584.5691	1668.7474	1205.1916
F6	3206.2187	3257.6666	1656.9126	1440.9406	8821.8510	2411.9727	1890.8204	1322.9937	1811.5445	3770.2810	2663.4595	2713.9477
F7	1372.0539	1288.7758	782.7713	663.4640	6866.7619	1437.7843	1011.0877	609.0990	1131.4985	1736.4851	1683.8658	1105.1710
F8	1705.4795	1618.2586	989.0674	758.6453	8018.2317	1703.7762	1208.4044	707.0991	1179.3074	1791.3102	1850.4034	1349.2233
F9	1648.6060	1651.0534	1003.9989	787.2143	7871.8631	1683.2607	1213.8018	706.4527	1227.0654	1776.2022	1956.8915	1337.1876
F10	2006.9759	1924.2230	1087.8128	844.9614	7858.7154	1727.0746	1286.4960	774.8843	1269.4878	2099.0346	2061.4072	1556.3573
F11	1429.5288	1345.2243	886.8715	648.1052	7671.3674	1591.8728	1076.9965	568.0270	1047.3130	1996.2238	1802.4607	1151.6052
F12	1707.7202	1715.7395	1056.6301	746.1166	8187.4304	1783.0518	1249.7590	738.4798	1189.7321	2169.5781	2011.4351	1447.6008
F13	1374.9731	1392.5722	916.9523	604.3327	7316.2702	1569.6265	964.5263	527.2179	1048.7610	1713.4424	1966.8551	1244.0637
F14	1495.8066	1510.9372	813.7888	630.3773	6341.5962	1057.0906	998.5398	584.8096	1055.0832	1223.2136	1551.7661	1311.9379
F15	920.7320	829.8522	539.6170	473.1657	6433.9494	1043.8119	831.0791	400.1807	886.7541	997.8084	1593.6538	1014.5658
F16	1377.2076	1422.0340	777.4971	601.3160	7099.0153	1383.3329	847.1459	500.7935	969.4762	1119.9581	1501.5904	1134.0044
F17	2240.5126	2367.8051	1179.4587	933.6498	7030.9006	1617.4542	1273.5408	856.2920	1363.3921	1960.5206	1786.7791	1711.5786
F18	1312.7182	1286.5012	762.6733	508.7210	6978.9989	1253.0395	923.6291	499.2580	1065.1539	1305.9613	1549.2926	1123.3055
F19	9161.5400	9250.5630	4060.2324	3705.0244	11,388.7078	4957.6448	4174.8165	3806.5568	4324.0079	8694.8813	5438.8193	7863.7214
F20	2315.3704	2213.6933	1065.6800	943.9558	6971.1951	1875.7916	2086.3267	1038.4659	1537.5500	2631.3454	2006.6912	2248.4016
F21	3899.9581	3845.7128	1697.2727	1524.9139	8402.9694	2783.3509	2117.3766	1609.1512	2042.7549	3562.7031	2991.9178	3465.8951
F22	4347.6517	4271.3248	1957.7470	1801.4333	8400.8835	2624.5653	2143.9029	1751.9575	2144.3526	3972.4113	3071.8435	3872.0125
F23	5243.3880	5095.2869	2343.7899	1945.2220	8023.0986	2870.1215	2572.9783	2019.1846	2638.2351	4842.5077	3447.0625	4812.5093
F24	5859.5971	5815.1403	2703.2262	2198.2537	9893.7107	3412.4870	2958.0299	2381.3381	2782.0250	5347.2745	3702.8456	5184.9546
F25	5234.0871	5325.2122	2328.7195	2169.1695	8902.0796	3287.3585	2627.7942	2089.5291	2537.6093	4462.6248	3028.2297	4218.4649
F26	6053.0398	6210.6052	2721.7291	2488.0499	8755.1673	3203.4136	2870.9227	2351.2427	2835.7235	5087.6332	3515.9223	5614.6762
F27	7363.3117	7377.8585	3176.2489	2678.6598	9494.8237	3698.5370	3358.6715	2957.4788	3373.3546	6099.7269	4054.0289	5826.5099
F28	6043.9795	6153.3206	2753.7941	2444.2012	8661.4358	3284.1057	2925.9665	2448.1554	2955.4936	5578.7806	3508.5614	4854.3631
F29	4402.0849	4255.1172	2049.2857	1816.3831	7972.2670	2373.5016	2140.2642	1814.0077	2068.3372	3796.2073	2672.3020	3387.8230
F30	11,255.9521	11,151.5382	4794.7383	3477.0287	11,783.6397	5529.2351	5132.7099	4466.0694	4932.9687	10,230.8298	6440.0952	10,042.3783

**Table 4:** Space complexity comparison between MACOA and the other 11 algorithms.

	Space Complexity		Space Complexity
MACOA	O(pop × dim)	TSA	O(pop × dim)
COA	O(pop × dim)	WOA	O(pop × dim)
SABO	O(pop × dim)	GWO	O(pop × dim)
WSO	O(pop × dim + Max × dim)	TLBO	O(pop × dim)
SCSO	O(pop × dim + Max)	GSA	O(Max × pop × dim)
GJO	O(pop × dim)	PSO	O(pop × dim + Max)

**Table 5:** Summary of the CEC-2017 test functions.

Name	No.	Functions	F_i_ (x*)	No.	Functions	F_i_ (x*)
Unimodal Functions	1	Shifted and Rotated Bent Cigar Function	100	3	Shifted and Rotated Zakharov Function	200
Simple Multimodal Functions	4	Shifted and Rotated Rosenbrock’s Function	300	8	Shifted and Rotated Non-Continuous Rastrigin’s Function	700
	5	Shifted and Rotated Rastrigin’s Function	400			
	6	Shifted and Rotated Expanded Scaffer’s F6 Function	500	9	Shifted and Rotated Levy Function	800
	7	Shifted and Rotated Lunacek Bi_Rastrigin Function	600	10	Shifted and Rotated Schwefel’s Function	900
Hybrid Functions	11	Hybrid Functions 1 (N = 3)	1000	16	Hybrid Functions 6 (N = 4)	1500
	12	Hybrid Functions 2 (N = 3)	1100	17	Hybrid Functions 6 (N = 5)	1600
	13	Hybrid Functions 3 (N = 3)	1200	18	Hybrid Functions 6 (N = 5)	1700
	14	Hybrid Functions 4 (N = 4)	1300	19	Hybrid Functions 6 (N = 5)	1800
	15	Hybrid Functions 5 (N = 4)	1400	20	Hybrid Functions 6 (N = 6)	1900
Composition Functions	21	Composition Functions 1 (N = 3)	2000	26	Composition Functions 6 (N = 5)	2500
	22	Composition Functions 2 (N = 3)	2100	27	Composition Functions 7 (N = 6)	2600
	23	Composition Functions 3 (N = 4)	2200	28	Composition Functions 8 (N = 6)	2700
	24	Composition Functions 4 (N = 4)	2300	29	Composition Functions 9 (N = 3)	2800
	25	Composition Functions 5 (N = 5)	2400	30	Composition Functions 10 (N = 3)	2900
Search Range: [−100, 100] ^dim^						

**Table 6:** Rank results comparing MACOA and the other 11 algorithms based on the CEC-2017 (the dimension m = 30).

	MACOA	COA	SABO	WSO	SCSO	GJO	TSA	WOA	GWO	TLBO	GSA	PSO
F1	2	11	7	5	6	8	9	12	4	1	3	10
F3	2	10	4	7	5	8	6	12	3	1	11	9
F4	1	11	8	7	5	6	9	12	4	3	2	10
F5	2	11	7	3	6	4	9	12	1	8	5	10
F6	2	10	7	4	6	3	8	11	1	12	5	9
F7	2	11	7	6	5	4	9	12	1	8	3	10
F8	3	10	7	2	6	5	9	12	1	11	4	8
F9	4	10	7	9	6	5	11	12	1	2	3	8
F10	1	10	9	4	5	6	7	11	3	12	2	8
F11	1	11	8	3	5	7	9	12	4	2	6	10
F12	2	11	6	7	5	8	9	12	4	1	3	10
F13	3	12	6	5	7	8	10	11	4	1	2	9
F14	3	11	9	4	5	7	10	12	6	2	8	1
F15	4	11	7	1	8	9	10	12	6	2	3	5
F16	3	11	9	1	5	4	8	12	2	6	7	10
F17	5	12	9	1	4	2	8	11	3	6	7	10
F18	2	11	9	3	8	6	10	12	7	4	5	1
F19	3	11	7	2	8	9	10	12	5	1	4	6
F20	3	10	8	1	5	4	6	12	2	9	7	11
F21	2	9	6	4	5	3	8	11	1	12	7	10
F22	1	11	4	7	5	6	9	12	2	3	8	10
F23	2	8	6	5	4	3	7	10	1	9	11	12
F24	1	11	5	8	4	3	7	9	2	12	6	10
F25	1	11	7	5	4	6	8	12	3	9	2	10
F26	1	11	8	6	5	3	9	12	2	4	7	10
F27	3	11	7	8	6	5	9	2	4	10	12	1
F28	1	12	9	7	6	8	11	3	5	10	4	2
F29	1	11	9	3	5	4	6	12	2	8	7	10
F30	2	11	7	3	6	8	10	12	5	1	4	9
Sum rank	63	311	209	131	160	162	251	319	89	170	158	239
Mean rank	2.172	10.724	7.207	4.517	5.517	5.586	8.655	11	3.069	5.862	5.448	8.241
Total rank	1	11	8	3	5	6	10	12	2	7	4	9

**Table 7:** Rank results comparing MACOA and the other 11 algorithms based on the CEC-2017 (the dimension m = 50).

	MACOA	COA	SABO	WSO	SCSO	GJO	TSA	WOA	GWO	TLBO	GSA	PSO
F1	1	11	6	8	5	7	9	12	4	2	3	10
F3	6	11	9	4	1	3	2	12	5	8	10	7
F4	1	11	7	8	5	6	9	12	4	2	3	10
F5	2	11	8	4	6	5	10	12	1	7	3	9
F6	2	9	7	4	6	3	10	11	1	12	5	8
F7	2	11	6	7	5	3	9	12	1	8	4	10
F8	2	11	8	4	6	5	10	12	1	7	3	9
F9	3	10	8	9	4	6	11	12	2	5	1	7
F10	1	10	9	4	5	6	7	11	3	12	2	8
F11	1	11	5	3	6	7	8	12	4	2	9	10
F12	2	12	6	8	5	7	9	11	4	1	3	10
F13	2	12	6	8	5	7	9	11	4	1	3	10
F14	2	12	8	7	5	6	10	11	4	1	9	3
F15	2	11	5	7	6	8	10	12	4	1	3	9
F16	1	12	8	4	7	6	9	11	2	5	3	10
F17	2	11	8	3	6	4	9	12	1	7	5	10
F18	1	11	9	4	6	8	10	12	5	2	3	7
F19	3	12	7	4	5	8	10	11	6	1	2	9
F20	3	10	9	1	5	4	7	12	2	11	6	8
F21	2	10	7	4	5	3	8	12	1	11	6	9
F22	1	9	8	3	5	6	7	11	2	12	4	10
F23	1	10	6	5	4	3	7	9	2	8	11	12
F24	1	12	5	8	3	4	6	11	2	9	7	10
F25	1	11	8	6	5	7	9	12	4	2	3	10
F26	1	11	7	5	4	3	9	12	2	8	6	10
F27	3	11	7	8	6	5	9	1	4	10	12	2
F28	3	12	11	8	7	9	10	2	5	4	6	1
F29	1	11	9	3	6	4	8	12	2	5	7	10
F30	2	11	8	5	6	7	9	12	4	1	3	10
Sum rank	55	317	215	156	150	160	250	315	86	165	145	248
Mean rank	1.897	10.931	7.414	5.379	5.172	5.517	8.621	10.862	2.966	5.690	5	8.552
Total rank	1	12	8	5	4	6	10	11	2	7	3	9

**Table 8:** Rank results comparing MACOA and the other 11 algorithms based on the CEC-2017 (the dimension m = 100).

	MACOA	COA	SABO	WSO	SCSO	GJO	TSA	WOA	GWO	TLBO	GSA	PSO
F1	1	11	6	8	4	9	5	12	3	2	7	10
F3	5	6	4	3	1	7	10	12	9	11	8	2
F4	1	11	7	8	4	5	6	12	3	2	9	10
F5	2	10	8	4	6	5	11	12	1	7	3	9
F6	2	9	8	4	6	5	10	11	1	12	3	7
F7	2	11	6	8	5	3	9	12	1	7	4	10
F8	2	11	8	4	6	5	10	12	1	7	3	9
F9	2	9	7	8	3	5	11	12	4	10	1	6
F10	1	10	9	4	5	6	7	11	3	12	2	8
F11	4	11	9	6	2	7	3	12	5	1	8	10
F12	1	11	5	7	4	6	9	12	3	2	8	10
F13	2	11	6	8	4	7	9	12	3	1	5	10
F14	1	11	9	6	3	8	7	12	4	2	5	10
F15	2	11	5	8	6	7	9	12	3	1	4	10
F16	1	11	9	4	6	5	8	12	2	3	7	10
F17	1	11	6	7	4	5	9	12	3	2	8	10
F18	2	11	9	6	5	7	8	12	4	1	3	10
F19	2	11	6	7	4	8	9	12	3	1	5	10
F20	2	10	9	1	4	6	7	12	3	11	5	8
F21	2	10	9	5	4	3	6	11	1	7	8	12
F22	1	10	9	3	5	6	7	11	4	12	2	8
F23	1	11	7	5	4	3	8	9	2	6	12	10
F24	1	12	8	6	3	4	7	10	2	5	11	9
F25	1	11	7	9	4	8	6	12	3	2	5	10
F26	1	11	9	5	4	3	6	12	2	8	7	10
F27	3	12	8	10	5	6	9	2	4	7	11	1
F28	3	12	9	11	6	8	7	2	5	4	10	1
F29	1	11	7	5	4	6	8	12	3	2	9	10
F30	1	11	5	7	4	6	9	12	3	2	8	10
Sum rank	51	308	214	177	125	169	230	319	88	150	181	250
Mean rank	1.759	10.621	7.379	6.103	4.310	5.828	7.931	11	3.034	5.172	6.241	8.621
Total rank	1	11	8	6	3	5	9	12	2	4	7	10

**Table 9:** Rank results of the ablation experiment for MACOA with CEC-2017 (dimension = 10).

	COA	COA + L	COA + N	COA + V	COA + L + N	COA + L + V	COA + N + V	MACOA
F1	8	7	5	3	6	1	4	2
F3	7	8	5	1	6	1	1	1
F4	8	7	6	1	5	4	2	3
F5	7	8	5	6	2	3	4	1
F6	8	7	6	3	5	2	4	1
F7	8	7	5	4	6	2	1	3
F8	7	8	2	3	1	5	6	4
F9	8	7	5	6	4	3	2	1
F10	8	7	6	4	5	1	3	2
F11	8	7	4	6	5	2	1	3
F12	8	7	6	4	5	3	2	1
F13	7	8	6	2	5	4	1	3
F14	7	5	8	4	6	1	3	2
F15	7	8	5	3	6	4	1	2
F16	8	7	3	6	4	1	5	2
F17	7	8	5	1	6	3	4	2
F18	5	8	6	3	7	4	1	2
F19	8	6	7	3	5	4	1	2
F20	7	8	6	3	5	2	1	4
F21	7	6	4	8	5	1	1	1
F22	8	7	1	3	6	5	2	4
F23	8	7	4	1	2	6	3	5
F24	5	8	1	7	4	6	2	2
F25	8	7	4	5	3	2	1	6
F26	7	8	4	1	5	6	3	1
F27	7	8	5	6	2	3	4	1
F28	7	8	3	6	4	2	1	5
F29	8	7	3	4	5	2	6	1
F30	7	8	6	3	5	4	1	2
Sum rank	213	212	136	110	135	87	71	69
Mean rank	7.345	7.310	4.690	3.793	4.655	3	2.448	2.379
Total rank	8	7	6	4	5	3	2	1

**Table 10:** Comparative analysis of the three-bar truss design problem.

Alg.	Best	Std	Mean	Median	Worst	FR	SR
MACOA	263.89584	0.00001	263.89585	263.89584	263.89588	2	10
COA	263.89604	0.00742	263.90185	263.89927	263.92995	0	0
SABO	263.89935	0.02207	263.92659	263.92195	264.00877	0	0
WSO	263.89587	0.00000	263.89587	263.89587	263.89587	0	0
SCSO	263.89585	0.00012	263.89592	263.89588	263.89662	0	0
GJO	263.89590	0.00052	263.89647	263.89634	263.89842	0	0
TSA	263.89593	0.00111	263.89761	263.89757	263.90125	0	0
SRS	263.89886	2.04942	264.70462	263.97716	270.91394	0	0
MPA	263.89584	0.00001	263.89585	263.89584	263.89587	8	18
TLBO	263.89584	0.00000	263.89584	263.89584	263.89584	0	70

**Table 11:** Comparative analysis of the tension or compression spring design problem.

Alg.	Best	Std	Mean	Median	Worst	FR	SR
MACOA	0.012612	0.000997	0.012863	0.012665	0.017698	98	98
COA	0.012687	0.002529	0.013488	0.013032	0.030455	0	0
SABO	0.012680	0.000156	0.012801	0.012740	0.013542	0	0
WSO	0.012665	0.000000	0.012665	0.012665	0.012665	0	100
SCSO	0.012665	0.000042	0.012708	0.012703	0.012843	0	2
GJO	0.012667	0.000020	0.012692	0.012687	0.012722	0	0
TSA	0.012670	0.000015	0.012695	0.012692	0.012738	0	0
SRS	0.012710	0.000110	0.012909	0.012885	0.013108	0	0
MPA	0.012665	0.000000	0.012665	0.012665	0.012665	0	100
TLBO	0.012665	0.000001	0.012666	0.012666	0.012670	0	16

**Table 12:** Results of the pressure vessel design problem experiments.

Alg.	Best	Std	Mean	Median	Worst	FR	SR
MACOA	5.885 × 10^3^	6.122 × 10^2^	6.335 × 10^3^	5.934 × 10^3^	7.319 × 10^3^	16	16
COA	7.141 × 10^3^	4.428 × 10^4^	6.047 × 10^4^	5.226 × 10^4^	2.036 × 10^5^	0	0
SABO	6.379 × 10^3^	4.770 × 10^2^	7.134 × 10^3^	7.036 × 10^3^	8.499 × 10^3^	0	0
WSO	5.885 × 10^3^	9.187 × 10^−13^	5.885 × 10^3^	5.885 × 10^3^	5.885 × 10^3^	0	0
SCSO	5.885 × 10^3^	5.175 × 10^2^	6.327 × 10^3^	6.023 × 10^3^	7.319 × 10^3^	0	0
GJO	5.886 × 10^3^	4.352	5.889 × 10^3^	5.889 × 10^3^	5.915 × 10^3^	0	0
TSA	5.889 × 10^3^	1.169 × 10^2^	5.931 × 10^3^	5.910 × 10^3^	6.716 × 10^3^	0	0
SRS	5.989 × 10^3^	1.741 × 10^2^	6.312 × 10^3^	6.335 × 10^3^	6.804 × 10^3^	0	0
MPA	5.885 × 10^3^	9.187 × 10^−13^	5.885 × 10^3^	5.885 × 10^3^	5.885 × 10^3^	0	100
TLBO	5.885 × 10^3^	2.282 × 10^−8^	5.885 × 10^3^	5.885 × 10^3^	5.885 × 10^3^	0	98

**Table 13:** Experimental results of the welded beam design problem.

Alg.	Best	Std	Mean	Median	Worst	FR	SR
MACOA	1.662	3.351 × 10^−2^	1.677	1.662	1.753	84	84
COA	1.735	2.118 × 10^−1^	2.427	2.488	2.773	0	0
SABO	1.691	5.034 × 10^−1^	1.981	1.785	3.502	0	0
WSO	1.662	0.000	1.662	1.662	1.662	100	100
SCSO	1.662	2.402 × 10^−4^	1.662	1.662	1.663	100	100
GJO	1.662	4.905 × 10^−4^	1.663	1.663	1.665	100	100
TSA	1.666	1.737 × 10^−3^	1.670	1.670	1.674	48	48
SRS	1.704	2.433 × 10^−2^	1.754	1.756	1.827	0	0
MPA	1.662	2.243 × 10^−16^	1.662	1.662	1.662	100	100
TLBO	1.662	2.243 × 10^−16^	1.662	1.662	1.662	100	100

**Table 14:** Experimental results of the speed reducer design problem.

Alg.	Best	Std	Mean	Median	Worst	FR	SR
MACOA	2.994 × 10^3^	2.679 × 10^1^	3.010 × 10^3^	3.006 × 10^3^	3.164 × 10^3^	46	46
COA	3.029 × 10^3^	4.527 × 10^97^	1.058 × 10^97^	3.281 × 10^3^	2.282 × 10^98^	0	0
SABO	3.220 × 10^3^	4.607 × 10^2^	4.360 × 10^3^	4.343 × 10^3^	5.277 × 10^3^	0	0
WSO	2.994 × 10^3^	0.000	2.994 × 10^3^	2.994 × 10^3^	2.994 × 10^3^	100	100
SCSO	2.995 × 10^3^	4.075	3.000 × 10^3^	3.001 × 10^3^	3.010 × 10^3^	0	0
GJO	2.995 × 10^3^	4.421	3.002 × 10^3^	3.001 × 10^3^	3.013 × 10^3^	0	0
TSA	3.006 × 10^3^	5.336	3.018 × 10^3^	3.019 × 10^3^	3.032 × 10^3^	0	0
SRS	3.041 × 10^3^	2.395 × 10^1^	3.081 × 10^3^	3.076 × 10^3^	3.149 × 10^3^	0	0
MPA	2.994 × 10^3^	0.000	2.994 × 10^3^	2.994 × 10^3^	2.994 × 10^3^	100	100
TLBO	2.994 × 10^3^	6.496 × 10^−14^	2.994 × 10^3^	2.994 × 10^3^	2.994 × 10^3^	100	100

**Table 15:** Experimental results of the gear train design problem.

Alg.	Best	Std	Mean	Median	Worst	FR	SR
MACOA	2.7009 × 10^−12^	6.8154 × 10^−9^	2.4924 × 10^−9^	8.8876 × 10^−10^	2.7265 × 10^−8^	0	92
COA	2.7009 × 10^−12^	4.5433 × 10^−7^	1.6367 × 10^−7^	1.8274 × 10^−8^	2.0226 × 10^−6^	0	82
SABO	2.7009 × 10^−12^	1.7358 × 10^−11^	8.2394 × 10^−12^	2.7009 × 10^−12^	1.1661 × 10^−10^	0	100
WSO	2.7009 × 10^−12^	6.1753 × 10^−12^	4.7386 × 10^−12^	2.7009 × 10^−12^	2.3078 × 10^−11^	0	100
SCSO	2.7009 × 10^−12^	4.2280 × 10^−10^	2.4751 × 10^−10^	2.3078 × 10^−11^	9.9216 × 10^−10^	0	100
GJO	2.7009 × 10^−12^	9.4329 × 10^−12^	8.8140 × 10^−12^	2.7009 × 10^−12^	2.3078 × 10^−11^	0	100
TSA	2.7009 × 10^−12^	3.2309 × 10^−10^	1.2633 × 10^−10^	2.7009 × 10^−12^	9.9216 × 10^−10^	0	100
SRS	2.7009 × 10^−12^	2.3366 × 10^−9^	2.1648 × 10^−9^	1.3616 × 10^−9^	8.7008 × 10^−9^	0	100
MPA	2.7009 × 10^−12^	2.8818 × 10^−12^	3.1084 × 10^−12^	2.7009 × 10^−12^	2.3078 × 10^−11^	0	100
TLBO	2.7009 × 10^−12^	1.3927 × 10^−10^	2.9418 × 10^−11^	2.7009 × 10^−12^	9.9216 × 10^−10^	0	100

**Table 16:** Results of the cantilever beam design problem experiments.

Alg.	Best	Std	Mean	Median	Worst	FR	SR
MACOA	1.33996	0.00000	1.33996	1.33996	1.33996	100	100
COA	1.35535	0.04447	1.42551	1.42357	1.52351	0	0
SABO	1.39712	0.06672	1.54173	1.53902	1.70340	0	0
WSO	1.33996	0.00000	1.33996	1.33996	1.33996	100	100
SCSO	1.33996	0.00000	1.33996	1.33996	1.33997	100	100
GJO	1.33996	0.00001	1.33997	1.33997	1.34001	98	98
TSA	1.34003	0.00012	1.34034	1.34034	1.34072	0	0
SRS	1.34537	0.01040	1.36089	1.35930	1.39957	0	0
MPA	1.33996	0.00000	1.33996	1.33996	1.33996	100	100
TLBO	1.33996	0.00000	1.33996	1.33996	1.33996	100	100

## Data Availability

The data generated from the analysis in this study can be found in this article. Any additional information required to reanalyze the data reported in this paper is available from the lead contact upon request.
